# POU2F1 Promotes Chemoresistance in Colorectal Cancer Cells via Attenuates the MDR2 Degradation Mediated by PPP1R11 Lactylation

**DOI:** 10.1002/advs.202522316

**Published:** 2026-03-07

**Authors:** Longzheng Xia, Jinguan Lin, Xianjie Jiang, Linda Oyang, Shiming Tan, Zongyao Ren, Qiu Peng, Qianjin Liao, Yujuan Zhou

**Affiliations:** ^1^ Hunan Key Laboratory of Cancer Metabolism Hunan Cancer Hospital and the Affiliated Cancer Hospital of Xiangya School of Medicine Central South University Changsha Hunan China; ^2^ Hunan Engineering Research Center of Tumor Organoid Technology and Application Public Service Platform of Tumor Organoids Technology 283 Tongzipo Road Changsha Hunan China; ^3^ Department of Oncology Hunan Provincial People's Hospital and the First Affiliated Hospital of Hunan normal university Hunan Normal University Health Science Center Changsha Hunan China

**Keywords:** colorectal cancer, lactate, MDR2, POU2F1, PPP1R11 lactylation

## Abstract

Acquired drug resistance continues to plague targeted therapies for colorectal cancer (CRC), with low chemosensitivity severely limiting treatment effectiveness. Our research reveals a key player in this challenge—POU2F1, a transcription factor known to drive tumor progression. We found this oncogene is markedly overexpressed in chemotherapy‐resistant CRC, where it undermines treatment by suppressing MDR2 protein degradation. Mechanistically, we discovered POU2F1 stimulates MCT4 expression, which in turn triggers the cytosolic lactate export, where decreases the level of PPP1R11 lactylation, a negative regulator of MDR2. Further experiments unveil that lactylation of PPP1R11, a critical post‐translational modification that normally stabilizes PPP1R11 and boosts its E3 ligase activity, promotes MDR2 ubiquitination and degradation, sensitizing cells to chemotherapy. Overall, our findings highlight the ability of POU2F1 in chemoresistance and regulating protein lactylation. Moreover, our work links lactylation to the exquisite regulation of the chemotherapy resistance by POU2F1, thus broadening the current knowledge of POU2F1 biological functions and the mechanism underlying target gene regulation.

## Graphical abstract

Schematic diagram of our study showing that highly expressed POU2F1 in colorectal cancer (CRC) can directly accelerate the cytosolic lactate export to decrease PPP1R11 lactylation at K59, thereby attenuating the E3 ligase enzyme activity and protein stability of PPP1R11, which inhibits the ubiquitination of MDR2 at K413 and K538 but increases the expression of MDR2 to drive chemoresistance in CRC.

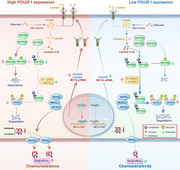

## 1 Introduction

Chemotherapy remains the primary treatment for intermediate/advanced colorectal cancer (CRC) and metastatic recurrence, demonstrating significant clinical success across multiple cancer types [1, 2, 3]. However, its efficacy is often limited by chemotherapy resistance, a major challenge in malignant tumors, including CRC [4]. The tumor microenvironment (TME), a complex ecosystem of pro‐ and antitumor factors‐, plays a crucial role in shaping treatment outcomes [2]. Within the TME, chemokines, proinflammatory cytokines, growth factors, and metabolic regulators critically contribute to chemoresistance in CRC [5]. Chemoresistance, particularly in combination therapies, has become a key focus in cancer research [6]. During the acquisition of drug resistance of cancer cells, various of molecules serve as pivotal regulators, including MDR1 (multidrug resistance 1) [7], MDR2 (multidrug resistance 2) [8], MRPs (multidrug resistance proteins) [9], etc.

The mechanism of drug efflux mediated by members of the ABC transporter protein superfamily is now considered to be a fundamental mechanism of drug resistance [7, 8]. The P‐glycoprotein (P‐gp) encoded by the P‐gp gene family that can mediate drug efflux is a major mechanism for the development of multidrug resistance [7]. It has been reported that the P‐gp gene family consists of two members, the MDR1 and the MDR2 gene [10]. More and more studies have identified the presence of abnormally high expression of MDR2 in drug‐resistant cells in a wide range of tumors, which may be involved in regulating their drug resistance process [11, 12, 13]. The function of MDR2 is to pump intracellular drugs out of the cell, altering the intracellular drug distribution and preventing the cytotoxic effects of the drug [8]. MDR2 transporter channels can exist in either an open or closed conformation with the absence of drug treatment [10]. MDR2‐transported channels are in a closed conformation, which may explain the lack of effect on cell proliferation, but studies are needed to confirm.

POU domain class 2 transcription factor 1 (POU2F1), also known as octamer transcription factor 1 (OCT1), was described as a regulator of drug resistance via promoting Warburg effect according to our previous study [14]. Meanwhile, there is also evidence showing that POU2F1 is necessary for oxaliplatin‐mediated DNA damage of colorectal cancer cell (CRC) [14]. Metabolic abnormalities are a hallmark of cancer cells, with Warburg effect the being one of the most metabolic alterations [15]. Interestingly, we also found that deletion of POU2F1 could accelerate the accumulation of cytosolic lactate and induces intracellular protein lactylation in pre‐experiment assay. Lactate acts as a metabolite of glycolysis to promote tumor progression by establishing an immunosuppressive TME [16]. Recently, lactate‐derived lactylation (La) of lysine (K) residue has been identified as an epigenetic modification that directly stimulates gene transcription from chromatin [17]. Protein lactylation was also reported to drive chemoresistance by facilitating DNA end resection and HR [18]. MRE11, a crucial homologous recombination (HR) protein, is lactylated at K673 by the CBP acetyltransferase in response to DNA damage and dependent on ATM phosphorylation of the latter [18]. Although it is evident that the regulation between POU2F1 and Warburg effect may induce the chemoresistance of CRC, the exact mechanisms underlying POU2F1‐dependent chemoresistance remain unclear.

Herin, we revealed that increasing intracellular lactate export by POU2F1 caused significant inhibition of PPP1R11 lactylation, which is critical for it‐mediated MDR2 K413 and K538 ubiquitination in CRC. Very interestingly, we identified that POU2F1 promoted MCT4 transcriptional expression to accelerate cytosolic lactate exporting, and then decreased the K59 lactylation of PPP1R11, which was essential for its stability and E3 ligand activity. These novel findings will be beneficial for understanding the molecular mechanisms involved in POU2F1‐mediated chemoresistance and explore the strategies targeting POU2F1 for potentiating MDR2 degradation against tumors.

## 2 Results

### 2.1 POU2F1 Induces Chemoresistance in a MDR2‐Dependent Manner

**FIGURE 1:**
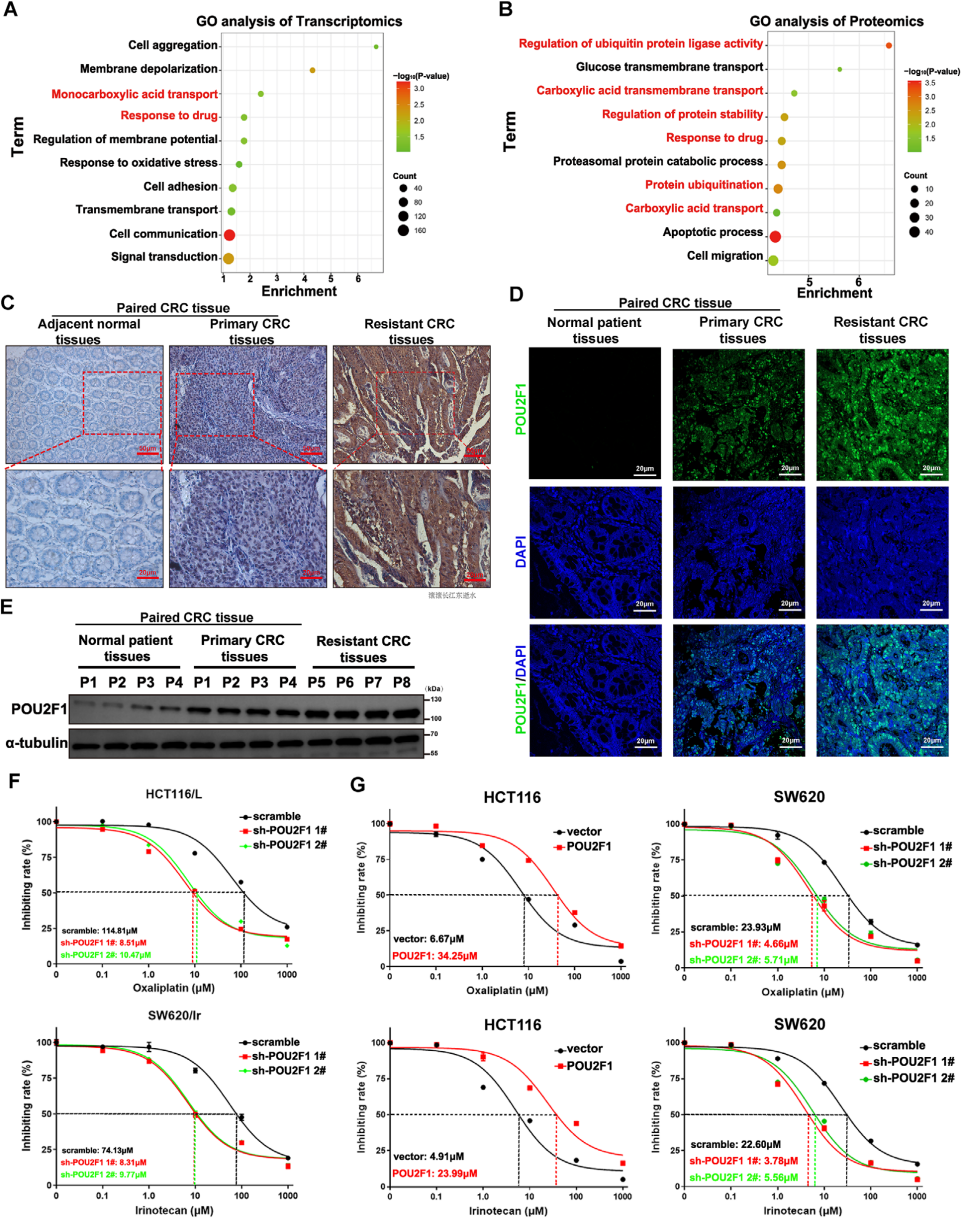
Up‐regulated POU2F1 is a predictive marker for chemotherapy resistance in colorectal cancer. (A,B) The top 10 enriched pathways under POU2F1 silencing were analyzed via GO enrichment analysis based on transcriptomics (A) and proteomics (B). (C,D) IHC staining (C) and immunofluorescence (IF) staining (D) of POU2F1 expression in 100 pairs of CRC tissues and matched para‐cancer tissues, and 10 chemo‐resistant CRC tissues (magnification x200, scale bars 50 µm, magnification x400, scale bars 20 µm). (E) Western blotting analysis of POU2F1 expression in 4 cases of normal tissues, primary CRC tissues and chemo‐resistant CRC tissues, respectively. (F) Survival curves for oxaliplatin‐resistant HCT116/L cells and irinotecan resistant SW620/IR cells after POU2F1 knockdown. (G) Survival curves for POU2F1 overexpressing/silencing CRC cells treated with oxaliplatin and irinotecan, respectively. Data are represented as the means ± standard deviation (SD). **p < 0.01; ***p < 0.001; ns, no significance.

To systematically elucidate the oncogenic role of POU2F1, we conducted quantitative proteomic profiling and transcriptome sequencing in POU2F1‐knockdown CRC cells. Bioinformatic analysis demonstrated significant enrichment of differentially expressed genes/proteins in drug response pathways (Figure 1A,B), implicating POU2F1 in chemotherapeutic sensitivity regulation. Interrogation of The Cancer Genome Atlas (TCGA)‐COAD and Gene Expression Omnibus (GEO) datasets (GSE3964, GSE69657, GSE72968) revealed POU2F1 overexpression in chemotherapy‐refractory CRC cohorts, with elevated expression correlating with adverse progression‐free survival (PFS) and overall survival (OS) (*p* < 0.05, Figure S1A–F). Immunohistochemical and immunoblot analyses confirmed POU2F1 upregulation in chemo‐resistant CRC specimens and cell line models compared to chemo‐naïve counterparts (*p* < 0.05, Figure 1C–E and Figure S1G–I). Functional validation studies demonstrated that POU2F1 overexpression protected CRC cells from oxaliplatin‐ and irinotecan‐induced cytotoxicity in dose‐ and time‐dependent manners (*p* < 0.05, Figure 1F,G and Figure S2A–E). Complementarily, genetic complementation in POU2F1‐depleted cells restored chemoresistance phenotypes (*p* < 0.05, Figure S2F). Collectively, these data establish POU2F1 as a key mediator of acquired chemoresistance in CRC.

**FIGURE 2:**
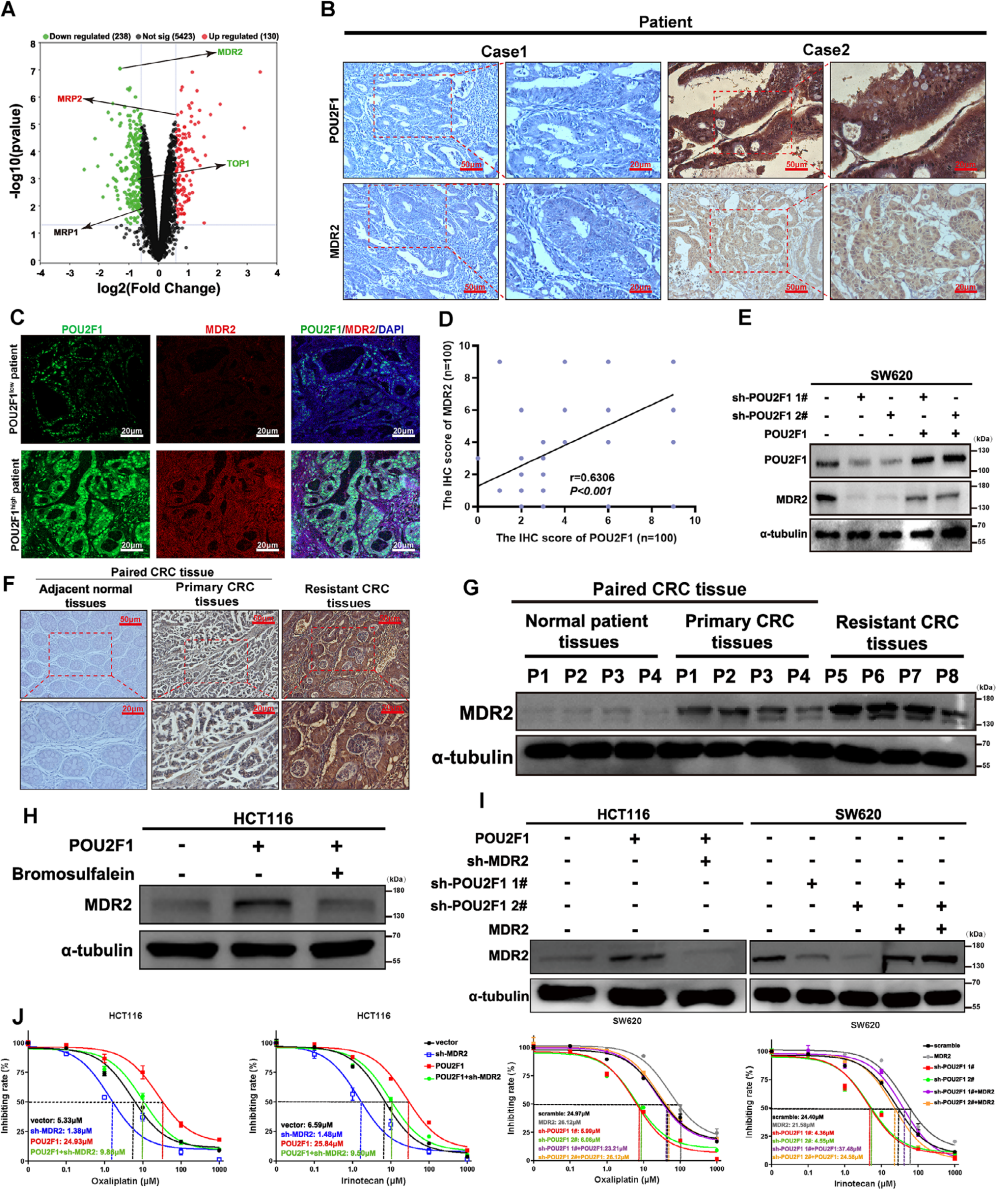
POU2F1 promotes chemotherapy resistance in a MDR2‐dependent manner. (A) Volcano plot analysis of differentially expressed proteins in POU2F1 silencing proteomics. (B, C) IHC staining (B) and IF staining (C) of POU2F1 and MDR2 expression in 100 cases of CRC tissues (magnification x200, scale bars 50 µm, magnification x400, scale bars 20 µm). (D) The correlation between the relative levels of POU2F1 and MDR2 in 100 paired of CRC tissues based on IHC staining scores. (E) Western blot analysis of the relative levels of MDR2 expression in the indicated groups of SW620 cells. (F, G) IHC staining (F) and Western blotting analysis (G) of MDR2 expression in 4 cases of normal tissues, primary CRC tissues and chemo‐resistant CRC tissues, respectively (magnification x200, scale bars 50 µm, magnification x400, scale bars 20 µm). (H) Western blotting analysis of MDR2 levels in POU2F1‐overexpressing CRC cells with 20 µM Bromosulfalein treatment for 48h. (I) Western blot analysis of the relative levels of MDR2 expression in the indicated groups of CRC cells. (J) Survival curves for the indicated groups of CRC cells treated with oxaliplatin and irinotecan, respectively. Data are represented as the means ± standard deviation (SD). **p < 0.01; ***p < 0.001; ns, no significance.

To delineate the mechanistic basis of POU2F1‐mediated chemoresistance, we analyzed proteomic data from POU2F1‐silenced CRC cells and identified MDR2, MRP2, and TOP2A‐known chemoresistance‐related proteins‐as differentially expressed (Figure 2A). Among these, MDR2 showed the most significant changes upon POU2F1 modulation (*p* < 0.05, Figure S3A,B). IHC confirmed a strong positive correlation between POU2F1 and MDR2 (*p* < 0.05, *r* = 0.6306, Figure 2B–D), and POU2F1 overexpression restored MDR2 levels in silenced cells (Figure 2E). Unlike MDR1 (a well‐known multidrug resistance gene), the role of MDR2 in chemoresistance was unclear. While MDR2 expression is higher in tumor tissues vs. adjacent tissues, its expression is most significant in chemotherapy resistant CRC (*p* < 0.05, Figure 2F,G and Figure S3C,D and Table 1). Survival analysis revealed High MDR2 correlated with poorer PFS and OS (*p* < 0.001, Figure S3E–G). Notably, MDR2 silencing/overexpression did not affect CRC growth (*p* < 0.05, Figure S4A–C), but its levels were higher in chemotherapy‐treated patients in TCGA cohort and GSE69657 database (*p* < 0.05, Figure S4D,E). Functionally, MDR2 overexpression reduced oxaliplatin‐ and irinotecan‐induced cell death (*p* < 0.01, Figure S4F). To confirm the role of MDR2 in POU2F1‐mediated resistance, we used shRNAs and the inhibitor Bromosulfalein to block MDR2 [19], both approaches reversed POU2F1‐induced chemoresistance (*p* < 0.01, Figure 2H–J and Figure S4G). These findings demonstrate that POU2F1 drives CRC chemoresistance by upregulating MDR2.

**TABLE 1:** Association of MDR2 expression and clinicopathological characteristics.

Variable		MDR2		
	*N*	High	Low	*p* Value
**Age** <60 ≥60	38 62	15 28	23 34	0.577
**Gender** Male Female	72 28	34 11	38 17	0.474
**Histology grading** Well differentiation Moderate differentiation Poor differentiation	28 39 33	15 24 11	13 15 22	0.053
**TNM stages** I II III IV	18 23 35 24	4 10 24 15	14 13 11 9	<0.001
**Metastasis** Yes No	22 78	14 22	8 56	0.002

### 2.2 Identification of PPP1R11 as a Negative Regulator of MDR2 Ubiquitination

**FIGURE 3:**
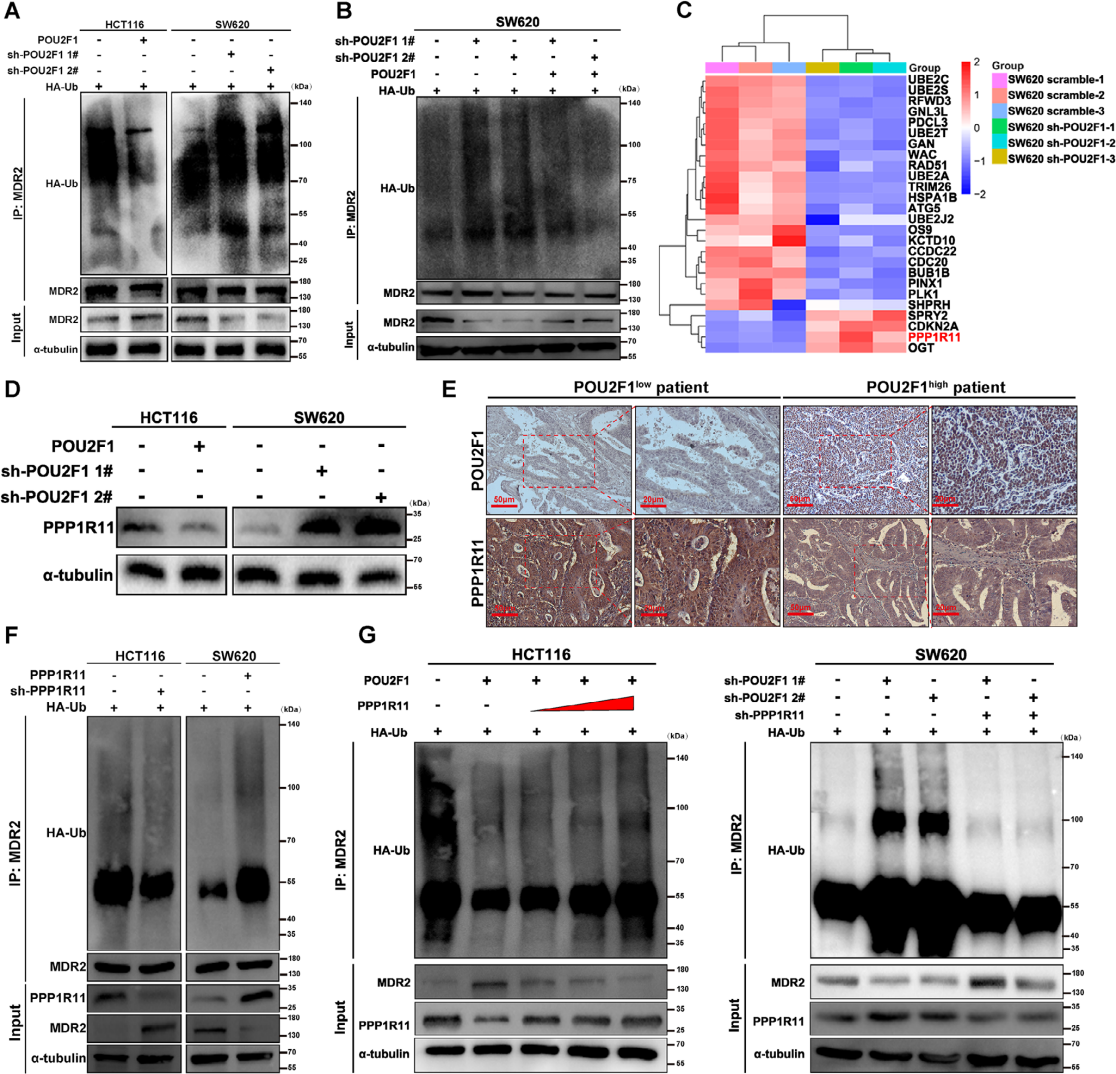
POU2F1 induces MDR2 expression via down‐regulating PPP1R11 expression. (A, B) Western blotting analysis of MDR2 ubiquitination in POU2F1 overexpressing/silencing CRC cells (A) and POU2F1 silencing SW620 cells treated with POU2F1 overexpression (B). (C) Hierarchical clustering analysis of the distribution of PPP1R11 between POU2F1 silencing and control SW620 cells. The DEGs were identified, based on absolute fold change ≥1.5 and *p*‐value <0.05. (D) Western blot analyses of the relative levels of PPP1R11 protein expression in the indicated cells. (E) IHC analysis of POU2F1 and PPP1R11 in CRC specimens (magnification x200, scale bars 50 µm, magnification x400, scale bars 20 µm). (F) Western blotting analysis of MDR2 ubiquitination in the indicated cells. (G) Western blotting analysis of the indicated proteins in CRC cells treated with PPP1R11 knockdown and overexpression for 48 h in the presence of POU2F1 inhibition/overexpression.

To elucidate how POU2F1 upregulates MDR2, we first observed that POU2F1 increased MDR2 protein levels without affecting its mRNA expression (*p* > 0.05, Figure S3A). Cycloheximide chase assays revealed that POU2F1 depletion shortened the half‐life of MDR2 (*p* < 0.01, Figure S4H–I), suggesting POU2F1 stabilizes MDR2 by inhibiting its degradation. Since our proteomic data linked POU2F1 silencing to protein ubiquitination pathways (Figure 1A), we hypothesized POU2F1 regulates MDR2 stability via ubiquitination. Co‐IP experiments confirmed that POU2F1 reduces MDR2 ubiquitination (Figure 3A,B), supporting its role in post‐translational stabilization. To identify the ubiquitin ligase responsible for MDR2 degradation, we screened our proteomic data and identified PPP1R11, a RING finger E3 ligase upregulated upon POU2F1 deletion (Figure 3C,D and S5A,B). Intriguingly, PPP1R11 was downregulated in CRC tissues based on TCGA database and IHC analysis (*p* < 0.05, Figure S5C–E and Table 2) and high PPP1R11 correlated with better survival (*p* < 0.001, Figure S5F), suggesting it acts as a tumor suppressor. Further analysis revealed a negative correlation between POU2F1 and PPP1R11 in CRC based on IHC analysis ((*p* < 0.05, *r* = −0.5451, Figure 3E and Figure S5G). Patients with POU2F1^low^/PPP1R11^high^ exhibited the longest OS and PFS, while POU2F1^high^/PPP1R11^low^ cases had the worst outcomes (*p* < 0.05, Figure S5H). These findings indicated PPP1R11 as a downstream effector of POU2F1 that governs MDR2 stability via ubiquitination.

**TABLE 2:** Association of PPP1R11 expression and clinicopathological characteristics.

Variable		PPP1R11		
	*N*	High	Low	*p* Value
**Age** <60 ≥60	38 62	24 40	14 22	0.891
**Gender** Male Female	72 28	43 21	29 7	0.153
**Histology grading** Well differentiation Moderate differentiation Poor differentiation	28 39 33	19 19 9	9 20 24	0.085
**TNM stages** I II III IV	18 23 35 24	13 7 17 6	5 16 18 18	<0.001
**Metastasis** Yes No	22 78	4 48	18 30	<0.001

Given prior evidence that the RING finger E3 ligase PPP1R11 mediates TLR2 ubiquitination [20], we investigated whether POU2F1 regulates MDR2 stability through PPP1R11. Notably, Co‐IP confirmed a physical interaction between PPP1R11 and MDR2 (Figure S6A), and an in vitro ubiquitination assay demonstrated PPP1R11 significantly enhances MDR2 polyubiquitination (Figure 3F). Next, domain‐deletion mutants localized the critical ubiquitination region to MDR2 residues 401–600 aa (Figure S6B,C). Systematic lysine‐to‐arginine mutagenesis identified K413 and K538 as essential sites for PPP1R11‐mediated ubiquitination and degradation (Figure S6D,E). Mutating either residue abolished MDR2 ubiquitination (Figure S6F). Moreover, PPP1R11 selectively catalyzed K48‐ and K63‐linked polyubiquitination of MDR2 (Figure S6G), which typically target proteins for proteasomal degradation or alter their function.

Taken together, PPP1R11 acts as a direct negative regulator of MDR2 by promoting its K48/K63‐linked ubiquitination at K413 and K538, thereby marking it for degradation.

### 2.3 POU2F1 Confers Chemoresistance by Restraining PPP1R11‐Mediated MDR2 Ubiquitination

**FIGURE 4:**
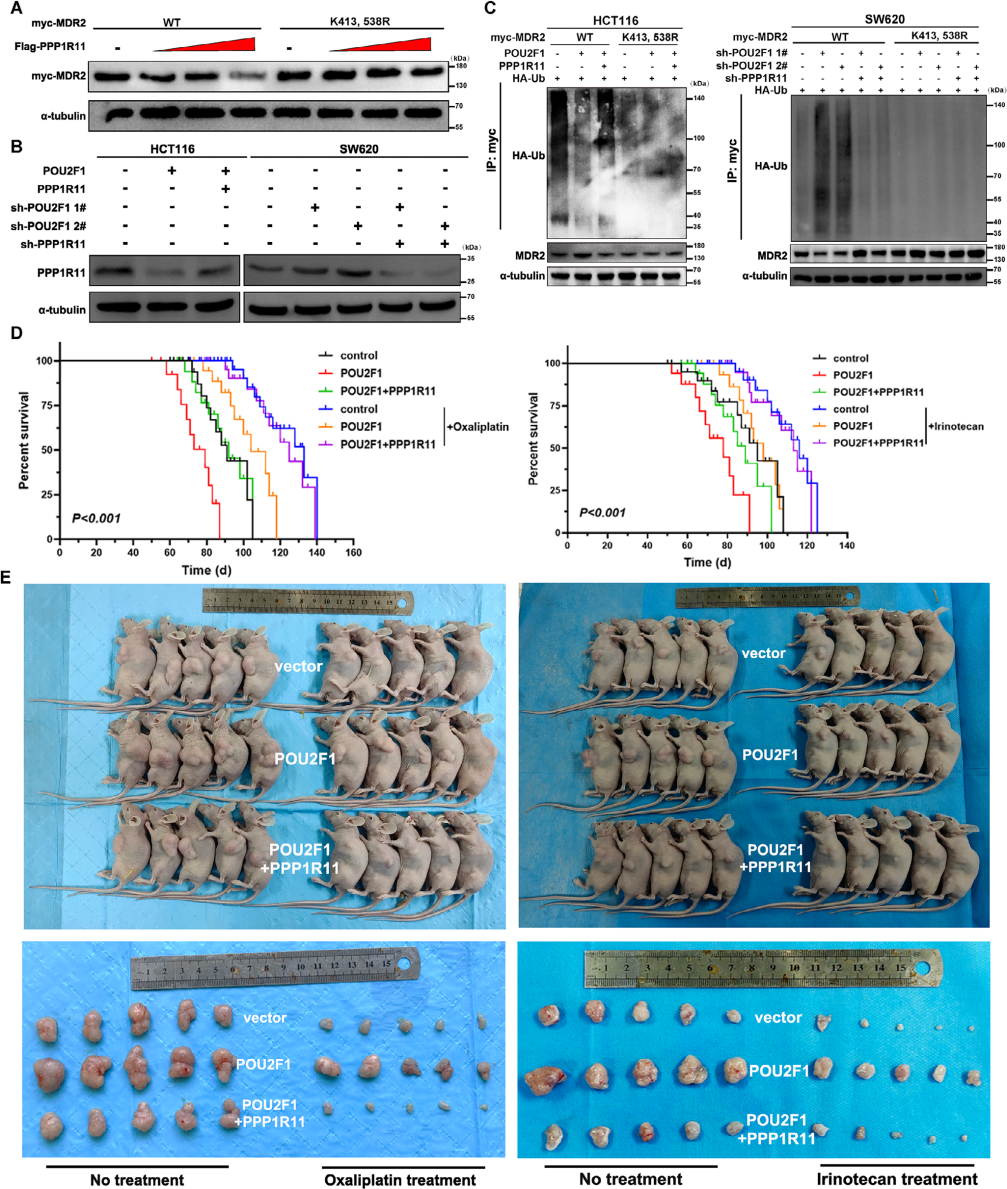
POU2F1 attenuated PPP1R11‐mediated MDR2 ubiquitination at K413 and K538 site in vivo and in vitro. (A) The myc‐MDR2 wild type (WT) or different MDR2 mutants (K413, K538R) was cotransfected with increasing concentrations of Flag‐PPP1R11 and the indicated proteins’ ubiquitination were detected by western blot. (B) Western blotting analysis of PPP1R11 expression in the indicated cells. (C) myc‐MDR2^WT^ or d MDR2^K413, K538R^ were cotransfected into CRC cells with PPP1R11 knockdown/overexpression in the presence of POU2F1 inhibition/overexpression. Cells were treated with MG132 before lysed. MDR2 was immunoprecipitated using an anti‐myc antibody. (D) Kaplan–Meier survival curves depicting overall survival (OS) of nude mice inoculated with LV‐vector, LV‐POU2F1, or LV‐POU2F1+PPP1R11 HCT116 cells with or without oxaliplatin and irinotecan (*n* = 10 per group), respectively. (E) Xenograft model in nude mice injected with cancer cells expressed with LV‐vector, LV‐POU2F1, or LV‐POU2F1+PPP1R11 HCT116 cells with or without oxaliplatin and irinotecan (*n* = 7 per group), respectively. Data are represented as the means ± standard deviation (SD). **p < 0.01; ***p < 0.001; ns, no significance.

Previous studies have demonstrated that PPP1R11 induces MDR2 ubiquitination in CRC cells, while POU2F1 overexpression suppresses PPP1R11 expression. To investigate whether POU2F1‐mediated PPP1R11 downregulation contributes to MDR2 ubiquitination and chemoresistance, we conducted a series of experiments. In vitro analyses revealed that PPP1R11 overexpression counteracted the inhibitory effect of POU2F1 on MDR2 ubiquitination at K413 and K538, whereas PPP1R11 silencing enhanced this suppression (Figures 3G and 4A–C). Furthermore, PPP1R11 overexpression reduced the proliferation of POU2F1‐overexpressing CRC cells (*p* < 0.05, Figure S7A), and restored the chemosensitivity of POU2F1‐overexpressing CRC cells to oxaliplatin and irinotecan (*p* < 0.05, Figure S7B). Notably, we performed an in vivo experiment and Kaplan–Meier analysis showed that HCT116‐POU2F1 mice had significantly shorter OS, but PPP1R11 coexpression extended survival (*p* < 0.05, Figure 4D). Tumor growth rate and weight were elevated in HCT116‐POU2F1 mice, whereas PPP1R11 overexpression attenuated these effects (*p* < 0.05, Figure 4E and Figure S7C,D). Besides, IHC analysis of MDR2 expression aligned with these observations (Figure S8).

These results collectively indicate that POU2F1 reduces PPP1R11‐mediated MDR2 ubiquitination, thereby promoting chemoresistance in CRC.

### 2.4 The Suppression of PPP1R11 Lactylation by POU2F1 Attenuates Its Expression and Enzymes Activity

Previous studies have shown that POU2F1 counteracts PPP1R11 expression to alleviate ubiquitin‐dependent degradation of MDR2. To elucidate the mechanism by which POU2F1 suppresses PPP1R11 expression in CRC, we focused on metabolic reprogramming, a known regulator of protein expression [21]. This approach was particularly relevant given our prior findings that POU2F1 promotes metabolic reprogramming to induce oxaliplatin resistance [14]. Intriguingly, our investigation revealed that POU2F1 significantly reduced cytosolic lactate levels (*p* < 0.05, FigureS9A,B), and cytosolic lactate concentrations showed positive correlations with both PPP1R11 expression (*r* = 0.5020, *p* < 0.05) MDR2 ubiquitination (*r* = 0.8828, *p* < 0.05) (Figure S9C–E). We further demonstrated that increased lactate availability (through 20 mM lactate supplementation, Syrosingopine treatment [a dual inhibitor of lactate transporters MCT1/MCT4], or rotenone) reversed POU2F1‐mediated PPP1R11 suppression [22]. Conversely, reducing cytosolic lactate (via LDH inhibitor RS6212 or LDHA siRNA) enhanced POU2F1's inhibitory effect on PPP1R11 expression (*p* < 0.05, Figure S10). These results collectively indicate that POU2F1 downregulates PPP1R11 expression by reducing cytosolic lactate accumulation in CRC cells.

**FIGURE 5:**
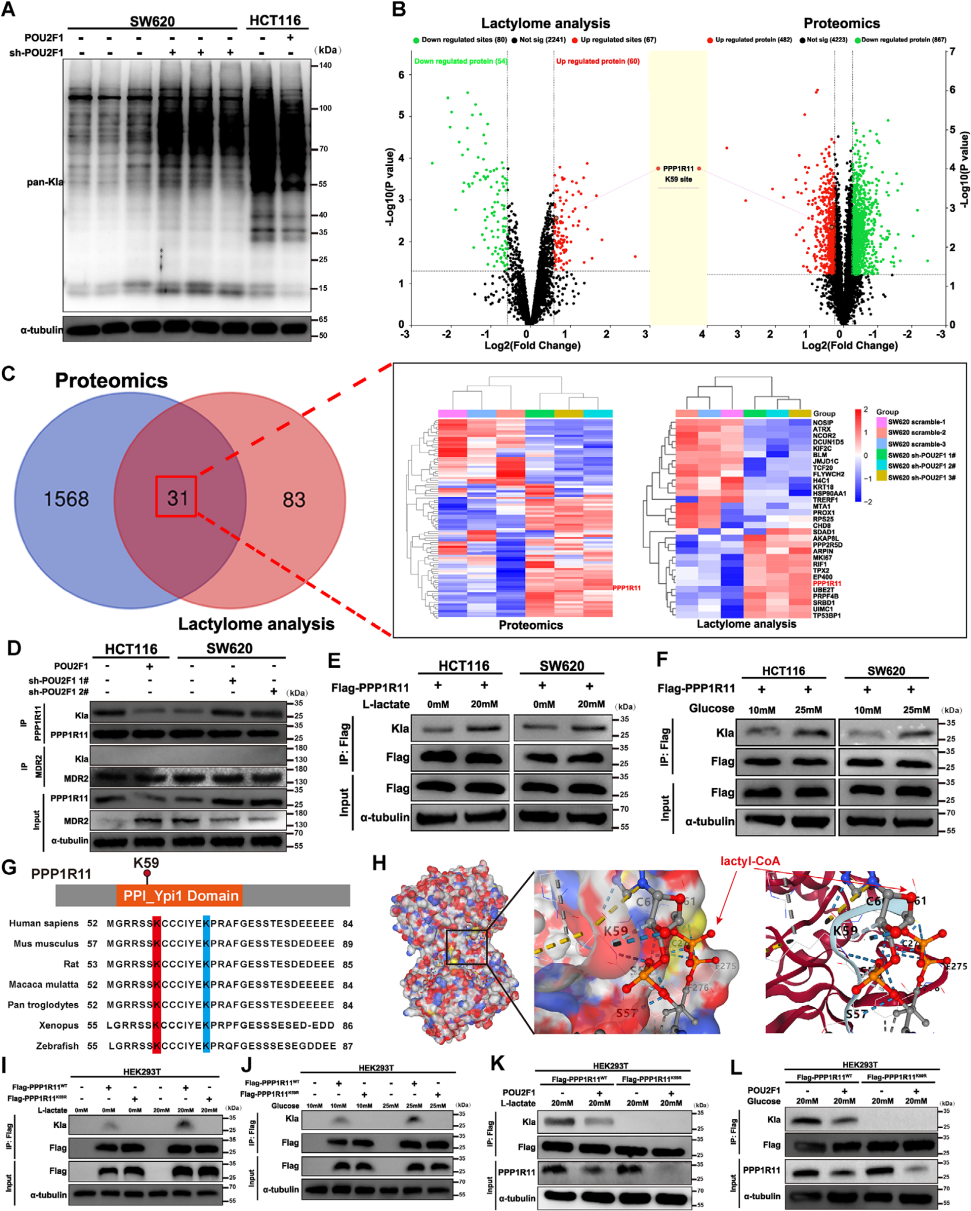
POU2F1 negatively regulates PPP1R11 lactylation at K59. (A) Kac levels were detected in SW620 cells transfected with scramble or POU2F1‐targted shRNAs and HCT116 cells with POU2F1 overexpression by immunoblotting. (B) Volcano plot showing differentially lactylated proteins and sites derived from proteomics and lactylome analysis. (C) Venn diagrams and Heatmap analysis of the proteomic expression levels of the differentially lactylated proteins between silencing POU2F1 cells and parental cells. (D) Western blotting analysis of PPP1R11 lactylation and expression in the indicated cells. (E, F) HCT116 and SW620 cells ectopically expressing Flag‐tagged PPP1R11 were stimulated with 20 mM lactate (E) or 10/25 mM glucose (F) for 24 h. Cell lysates were subjected to immunoprecipitation (IP) with Flag beads or Kla antibody conjugated agarose. Inputs and eluates were analyzed by immunoblots. (G) Additional mutants for fine mapping the lysine lactylation site within PPP1R11 (1–125aa). Sequence alignment of the region marked in orange (50–89aa) suggested conservation in mammalian species. (H) Interactions between lactyl‐CoA and PPP1R11are displayed via molecular docking analysis. S57, K59, C60, and C61 in PPP1R11 and the lactyl‐CoA molecule are shown as sticks (red or gray). The indicated hydrogen bond is shown as a blue or yellow dotted line. (I,J) HEK293T cells ectopically expressing Flag‐tagged PPP1R11 and Flag‐tagged PPP1R11‐K59R were stimulated with 20 mM lactate (I) or 10/25 mM glucose (J) for 24 h as described in (E,F). (K, L) PPP1R11‐WT and K59R stable cell lines ectopically expressing POU2F1 were treated with 20 mM lactate (K) or 25 mM glucose (L) for 24 h and then PPP1R11 lactylation was assessed by the immunoblots. Data are represented as the means ± standard deviation (SD). *p < 0.05; **p < 0.01; ***p < 0.001; ns, no significance.

Having demonstrated that POU2F1 reduces PPP1R11 expression by decreasing cytosolic lactate levels, we sought to elucidate the molecular mechanism linking low cytosolic lactate to PPP1R11 downregulation in CRC. Recent studies highlight lactylation as a crucial post‐translational modification through which cytosolic lactate regulates protein stability, enzymatic activity, and protein–protein interactions. In this study, our investigation yielded that global lactylation (Kla) levels were significantly elevated in POU2F1‐silenced cells compared to controls (Figure 5A). Integrated lactylome and proteomic analyses revealed increased PPP1R11 expression and lactylation in POU2F1‐silenced SW620 cells (Figure 5B,C), and ex vivo validation confirmed PPP1R11 lactylation from lactylome data (Figure 5D–F). Only one conserved lactylation site (K59) was identified in PPP1R1 (Figure 5B), which displayed remarkable evolutionary conservation Zebrafish to Homo sapiens (Figure 5G). Next, we generated a PPP1R11 K59R mutant and observed that K59R mutation substantially reduced lactylation levels under lactate or glucose stimulation (Figure 5I–L), indicating that lactylation mainly occurs at K59 site of PPP1R11.

To further elucidate how lactylation mediates POU2F1‐induced PPP1R11 inhibition, we conducted a series of functional experiments. First, we observed that POU2F1 significantly shortened the half‐life of PPP1R11, an effect reversed by lactate or high glucose treatment (Figure S11A,B), and enhanced PPP1R11 ubiquitination, which was attenuated by POU2F1 inhibition, lactate, or high glucose treatment (Figure S11C–F). And then, immunoblotting showed POU2F1 markedly decreased the half‐life of PPP1R11^K59R^ mutant but not wild‐type (WT) PPP1R11 under lactate treatment (Figure S11G–H), demonstrating lactylation's essential role in protein stability. Additionally, the PPP1R11^K59R^ mutant exhibited stronger ubiquitination than WT following lactate or high glucose treatment, with POU2F1 overexpression further enhancing this effect (Figure S11I–M). Coincidently, we first found that PPP1R11WT showed significantly higher E3 ligase activity than the K59R mutant under lactate or high glucose conditions (Figure S12A,B), consistent with previous reports [23]. Furthermore, POU2F1 attenuated PPP1R11WT E3 ligase activity during lactate treatment but had no effect on the K59R mutant (Figure S12C). Collectively, these results demonstrate that POU2F1 promotes PPP1R11 inhibition by impairing its lactylation, thereby reducing both protein stability and E3 ligase activity.

### 2.5 POU2F1 Restrains PPP1R11‐Mediated MDR2 Ubiquitination by Counteracting Its Lactylation

**FIGURE 6:**
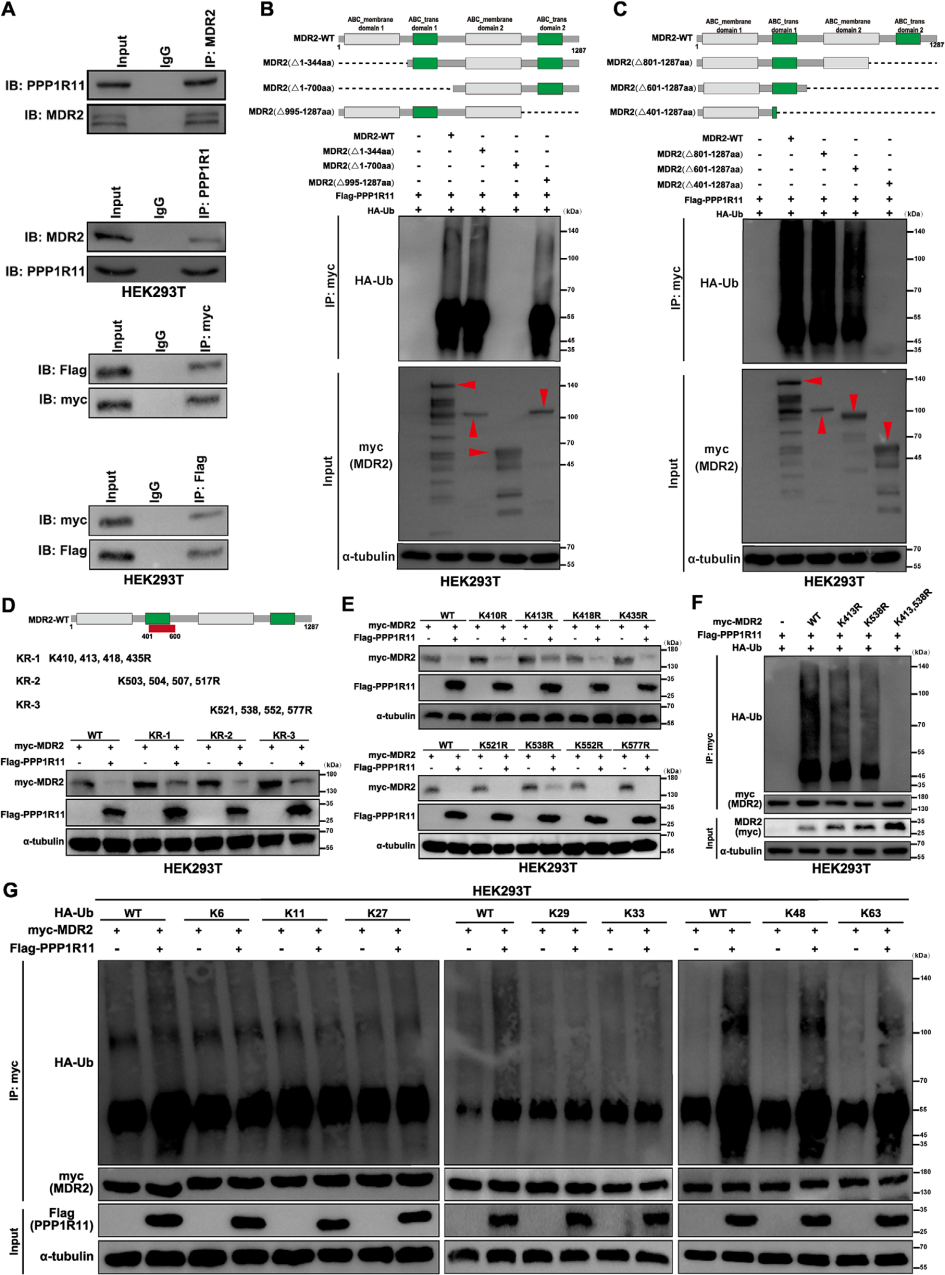
POU2F1 alleviates PPP1R11‐mediated MDR2 ubiquitination via restraining PPP1R11 lactylation. (A–D) Immunoblots for PPP1R11 lactylation and MDR2 ubiquitination in HEK293T cells expressing a vector control, Flag‐tagged PPP1R11, Flag‐tagged PPP1R11‐K59R, myc‐MDR2, and HA‐ubiquitin in the absence or presence of 20 mM lactate (A, C) or 10/25 mM glucose (B, D) for 24 h. (E) Western blot analyses for PPP1R11 lactylation and MDR2 ubiquitination in HEK293T si‐LDHA cells expressing a vector control, Flag‐tagged PPP1R11, Flag‐tagged PPP1R11‐K59R, myc‐MDR2, and HA‐ubiquitin in the presence of 10 mM glucose for 24 h. (F, G) Immunoblots for PPP1R11 lactylation and MDR2 ubiquitination in POU2F1‐overexpressing HCT116 cells expressing a vector control, Flag‐tagged PPP1R11, Flag‐tagged PPP1R11‐K59R, myc‐MDR2, and HA‐ubiquitin in the absence or presence of 20 mM lactate (F) or 10/25 mM glucose (G) for 24 h.

Given the critical role of PPP1R11 lactylation at K59 in regulating its stability and E3 ligase activity, we first examined how lactate and glucose influence MDR2 ubiquitination. Treatment with high‐dose lactate or glucose robustly increased MDR2 ubiquitination in cells expressing wild‐type PPP1R11 (PPP1R11^WT^), whereas the K59R mutant (PPP1R11^K59R^) only partially recapitulated this effect (Figure 6A,B). Consistent with this, PPP1R11‐mediated MDR2 ubiquitination was markedly weaker under low‐dose lactate or glucose conditions (Figure 6C,D). A similar pattern of the ectopic expressing LDHA on MDR2 ubiquitination was observed the different groups of PPP1R11^K59R^ and PPP1R11^WT^ CRC cells (Figure 6E), suggesting that PPP1R11 lactylation is critical for it‐mediated MDR2 ubiquitination. To test whether POU2F1 restricts MDR2 ubiquitination by modulating PPP1R11 lactylation, we performed in vitro ubiquitination assays and the results showed that PPP1R11^WT^ fully rescued MDR2 ubiquitination despite POU2F1's inhibitory effects, whereas PPP1R11^K59R^ only partially restored ubiquitination, even in the presence of lactate (Figure 6F,G), implying that POU2F1 dampens MDR2 ubiquitination by specifically interfering with PPP1R11 lactylation. We next asked whether PPP1R11 lactylation contributes to POU2F1‐driven malignancy in CRC. Indeed, PPP1R11^WT^ enhanced growth suppression and sensitized cells to oxaliplatin and irinotecan (Figure S12D,E). Conversely, while PPP1R11^WT^ completely counteracted POU2F1‐induced chemoresistance, PPP1R11^K59R^ had only a partial effect (*p* < 0.05, Figure S12F). Collectively, these data support a model in which POU2F1 inhibits PPP1R11‐mediated MDR2 ubiquitination by blocking lactylation at K59.

### 2.6 MCT4‐Mediated Lactate Secretion Facilitated by POU2F1 Contributed to the Reduction of PPP1R11 Lactylation

**FIGURE 7:**
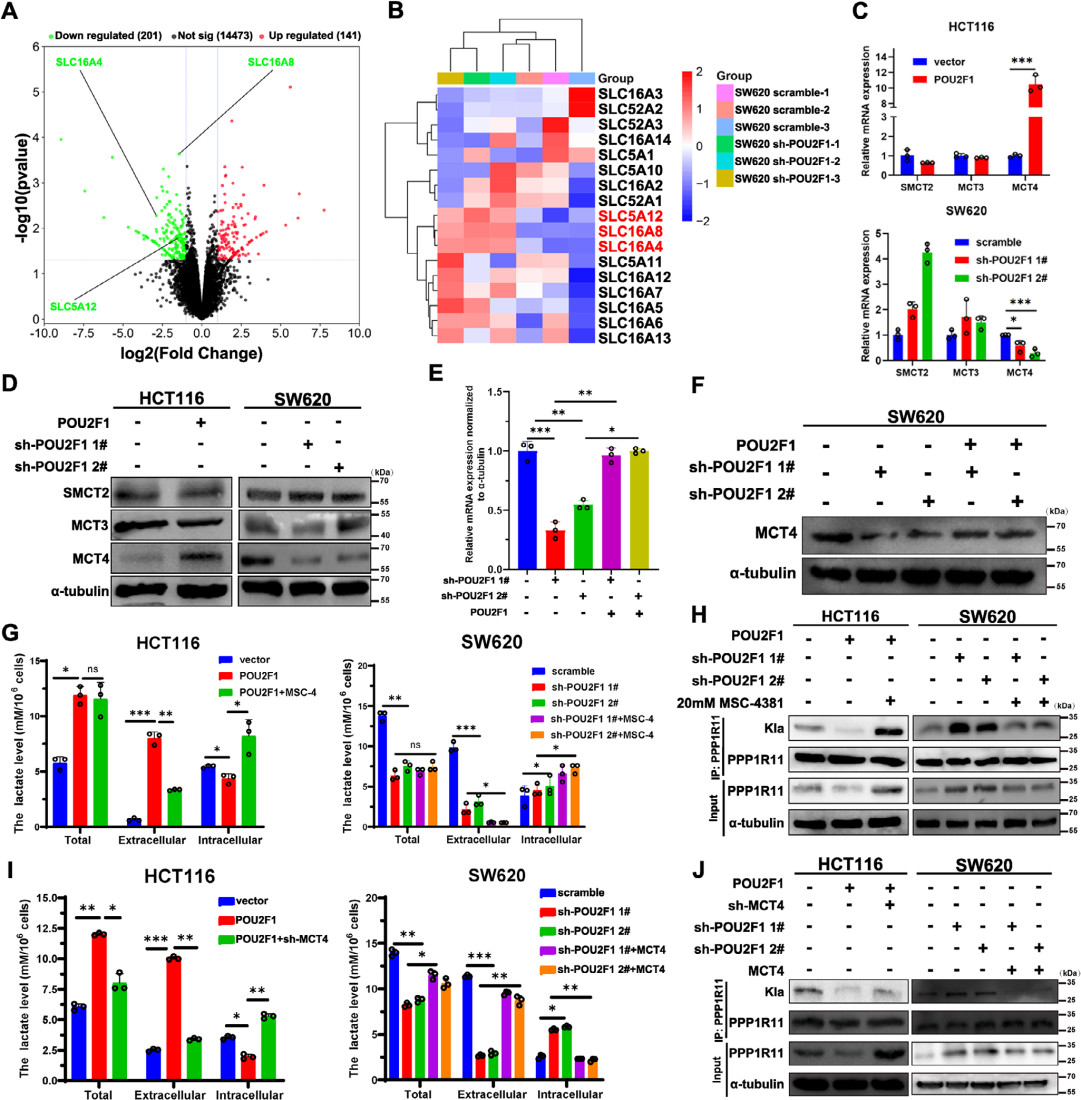
POU2F1 restrains PPP1R11 lactylation via up‐regulating MCT4 expression. (A, B) Volcano plot (A) and Heatmap (B) showing the transcriptomic expression of genes involving the production and transport of lactate according to silencing POU2F1 cells and parental cells. The three columns of histograms on the right show the results of differential expression analysis of each gene in indicated comparisons on the top of each column. (C, D) The mRNA (C) and protein (D) levels of SMCT2, MCT3, and MCT4 in POU2F1 silencing/ overexpressing CRC cells. (E, F) The mRNA (E) and protein (F) levels of SMCT2, MCT3, and MCT4 in POU2F1 silencing CRC cells rescued with POU2F1 overexpression. (G, H) Quantification of intracellular lactate (G) and protein level of PPP1R11 lactylation (H) in the indicated cells with or without 20 mM MSC‐4381 treatment. (I, J) Quantification of intracellular lactate (I) and protein level of PPP1R11 lactylation (J) in the indicated cells with MCT4 silencing/ overexpression. Data are represented as the means ± standard deviation (SD). *p < 0.05; **p < 0.01; ***p < 0.001; ns, no significance.

Having shown that POU2F1 reduces PPP1R11 lactylation by lowering cytosolic lactate levels, we sought to determine how POU2F1 achieves this effect. Intriguingly, while POU2F1 overexpression increased total lactate production, it paradoxically decreased intracellular lactate content (Figure 5A), suggesting enhanced lactate export. Since monocarboxylate transporters (MCT1‐4) play key roles in lactate shuttling between glycolytic and oxidative cells, we revisited our earlier transcriptomic data and revealed consistent downregulation of MCT4, MCT3, and SMCT2 in POU2F1‐silenced CRC cells (Figure 7A,B). While most studies focus on MCT1‐mediated lactate import, our findings highlight MCT4's unique role—among all MCT family members, only MCT4 showed dramatic reduction at both mRNA and protein levels upon POU2F1 knockdown, an effect reversible by POU2F1 re‐expression (*p* < 0.05, Figure 7C–F and Figure S13A,B). Therefore, we selected it for further analysis. Clinically, high MCT4 expression correlated strongly with poorer PFS and OS in CRC patients (*p* < 0.001, Figure S13C–E). Notably, patients with POU2F1^high^/MCT4^high^ tumors fared worst, while those with POU2F1^low^/MCT4^low^ profiles showed best outcomes (*p* < 0.001, Figure S13F). Experimentally, MCT4 deletion increased cytosolic lactate accumulation, which MCT4 overexpression rescued (*p* < 0.05, Figure S14A). To test the role of MCT4 in PPP1R11 lactylation, we found that MCT4 inhibition reduced lactylation even under high lactate/glucose conditions (Figure S14B–E). Conversely, MCT4 overexpression blunted the lactylation‐enhancing effects of these treatments (Figure S14B–E). The critical question remained: does MCT4 mediate POU2F1's effects? Indeed, MCT4 inhibitor MSC‐4381 elevated both intracellular lactate and PPP1R11 lactylation during high glucose treatment (*p* < 0.05, Figure 7G,H). Moreover, MCT4 silencing largely abolished the effect of POU2F1 to reduce cytosolic lactate and PPP1R11 lactylation under these conditions (*p* < 0.05, Figure 7I,J and Figure S14F). Together, these findings establish MCT4‐driven lactate export as the key mechanism through which POU2F1 suppresses PPP1R11 lactylation.

### 2.7 POU2F1 Transcriptionally Activated MCT4 Expression in Colorectal Cancer

**FIGURE 8:**
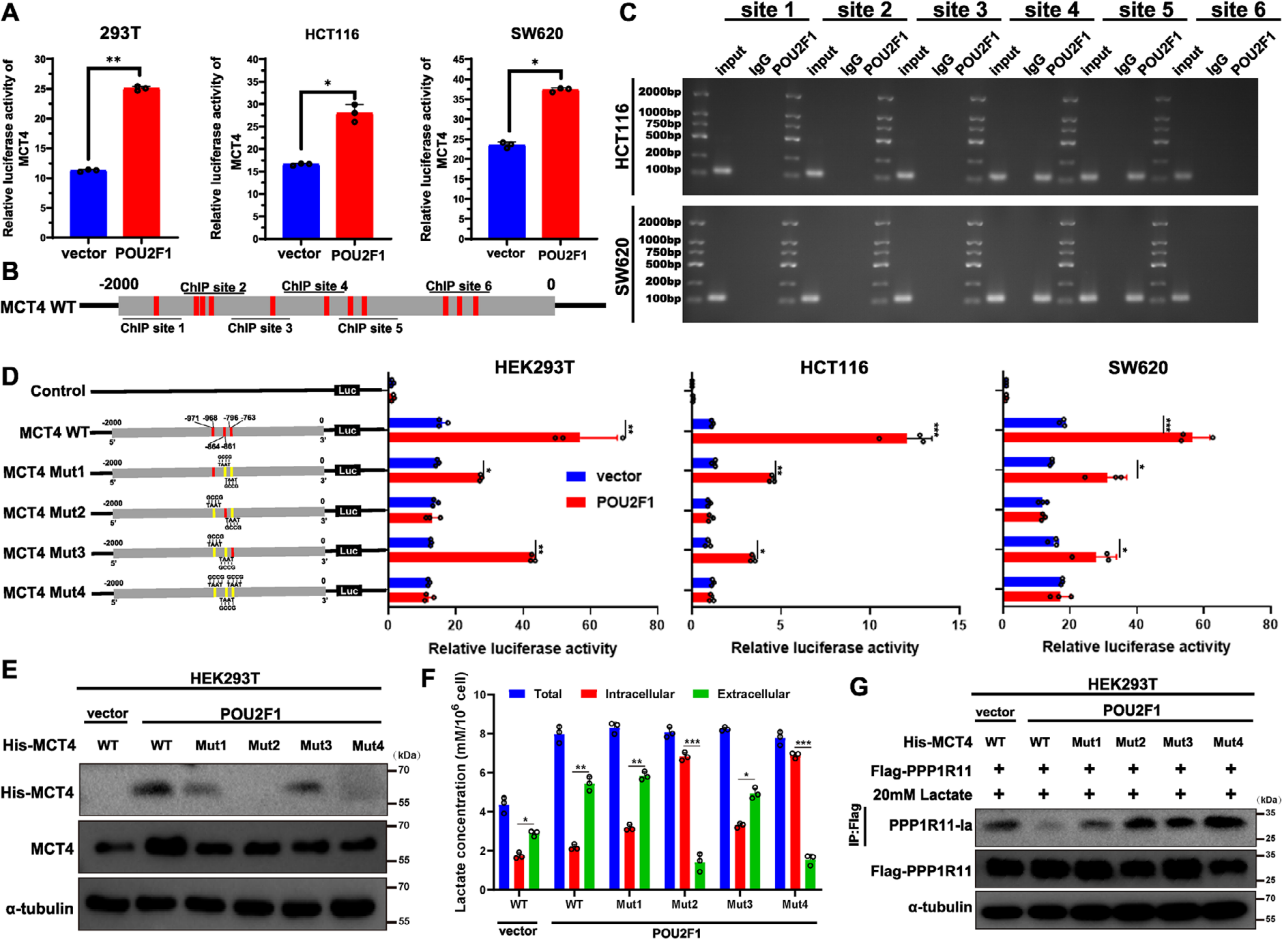
POU2F1 up‐regulates MCT4 expression via promoting its transcriptional activity. (A) Luciferase reporter assays indicated that POU2F1 enhanced the MCT4 promoter‐controlled luciferase expression in the indicated cells. (B) Schematics of the POU2F1 putative binding sites in the 5′ upstream regions of the MCT4 promoter (−2000 bp ∼ +50 bp). (C) Chromatin immunoprecipitation assay indicated that POU2F1 bound to the MCT4 promoter at sites 3, and 4. (D) Luciferase reporter assays determine POU2F1 bound to the MCT4 promoter at −971∼ −968 bp and −796∼−793 bp. (E, F) Western blotting and lactate assay to determine the influence of MCT4 promoter mutants on the protein levels of MCT4 (E) and quantification of cytosolic lactate (F) in the indicated cells, respectively. (G) Western blotting for PPP1R11 lactylation in HEK293T POU2F1 expressing HEK293T cells expressing Flag‐tagged PPP1R11, and His‐MCT4 in the presence of 20 mM lactate for 24 h. Data are represented as the means ± standard deviation (SD). *p < 0.05; **p < 0.01; ***p < 0.001; ns, no significance.

To understand how POU2F1 upregulates MCT4 to reduce intracellular lactate accumulation in CRC, we first confirmed that POU2F1 deletion markedly reduced MCT4 mRNA levels. This prompted us to investigate whether POU2F1 directly regulates MCT4 transcription. Using luciferase reporter assays, we identified strong promoter activity within the 2000bp region upstream of the MCT4 transcription initiation site (*p* < 0.05, Figure 8A). Bioinformatic analysis revealed eleven potential POU2F1 binding sites (TAAT core sequence) in the MCT4 promoter (Figure 8B). ChIP‐qPCR in HEK293T, HCT116, and SW620 cells showed specific POU2F1 binding to sites 6–8 (−1000 to −780bp region), with no signal detected in IgG controls (Figure 8C). To pinpoint the critical binding sites, we generated MCT4 promoter mutants, luciferase assays demonstrated that POU2F1 overexpression significantly enhanced wild‐type (WT) MCT4 promoter activity. Intriguingly, mutations at sites 2 and 4 (−971∼−968bp and −796∼−793bp) completely abolished this activation, while mutations at sites 1 and 3 had partial effects (*p* < 0.05, Figure 8D). Consistent with these findings, western blot analysis showed that only MCT4‐mut2 and ‐mut4 impaired POU2F1's ability to promote MCT4 expression and lactate export (Figure 8E,F). As expected, PPP1R11 lactylation showed an inverse pattern across these mutants (Figure 8G). These results clearly demonstrate that POU2F1 binds directly to two specific TAAT motifs in the MCT4 promoter to drive transcription, thereby enhancing lactate efflux and ultimately reducing PPP1R11 lactylation.

## 3 Discussion

Our previous work demonstrated that elevated POU2F1 in CRC correlates with poor prognosis and promotes oxaliplatin resistance, highlighting its potential as a therapeutic target [14]. Chemoresistance often involves ATP‐binding cassette (ABC) transporters, which mediate drug efflux [24, 25]. While ABC transporter‐targeted therapies show clinical promise, optimizing their inhibitors requires deeper insights into their regulatory mechanisms in tumors [10]. Here, we reveal that POU2F1 deficiency sensitizes CRC cells to oxaliplatin and irinotecan by enhancing MDR2 ubiquitination and degradation, coinciding with PPP1R11 upregulation. MDR2, a key member of ABC transporter, is a well‐established regulator of multidrug resistance in cancers [26, 27]. Notably, our findings align with recent studies by Awoniyi et al. [28] and Shriki et al. [29]. Mechanistically, POU2F1 suppresses PPP1R11 expression and inhibits MDR2 ubiquitination, effects reversible upon PPP1R11 overexpression in vitro. These results establish POU2F1 as a regulator of MDR2 stability via PPP1R11‐mediated ubiquitination, proposing POU2F1 inhibition as a novel strategy to overcome CRC chemoresistance.

Tumor hypoxia and oncogenes drive glycolytic metabolism, resulting in excessive lactate production and cytosolic acidification [30]. Monocarboxylate transporters (MCT1‐4), particularly MCT1 and MCT4, play distinct roles in lactate homeostasis [31]. While MCT1 imports lactate to fuel tumor growth, MCT4 predominantly expressed under hypoxic conditions—primarily exports lactate when intracellular levels rise [31, 32, 33]. Notably, MCT inhibition‐induced lactate accumulation promotes chemoresistance [34, 35, 36]. Our study reveals that POU2F1 specifically upregulates MCT4 (but not MCT1) by enhancing its promoter activity. Importantly, MCT4 inhibition reversed POU2F1‐mediated effects on both lactate accumulation and PPP1R11 expression. These findings demonstrate that intracellular lactate trapping can synergize with POU2F1's impact on glycolytic regulation.

Lactate, a glycolysis byproduct, serves as both a metabolic substrate and signaling molecule in tumor biology [37, 38]. In the TME, lactate promotes immunosuppression and tumor progression [39, 40, 41, 42, 43]. A key mechanism is lactylation (Kla), an epigenetic modification where lactate modifies lysine residues to regulate cellular processes [17]. This modification, catalyzed via lactyl‐CoA formation, influences tumor cell behaviors including drug resistance by modulating signaling pathways and gene expression [35, 44, 45, 46, 47, 48]. As well known, lactylation was initially found to mainly occur on histones, but in recent years, research has also expanded to nonhistones, including proteins in the cytoplasm and nucleus, including membrane protein. For membrane proteins, lysine residues on their intracellular domains are the main sites for lactylation. These areas are exposed in the cytoplasm and can come into contact with “donor” molecules produced by lactate [49]. Membrane proteins, especially their cytoplasmic parts, can undergo lactylation modification, and this modification is one of the important mechanisms for metabolites to directly and rapidly regulate membrane protein function. Some studies have provided specific evidence of membrane protein lactylation. For example, studies have shown that EGFR can be lactate modified, which in turn affects its dimerization, autophosphorylation, and activation of downstream signaling pathways such as MAPK and PI3K/AKT, ultimately affecting tumor cell proliferation [50]. However, our study reveals that POU2F1 reduces global protein lactylation in CRC cells, particularly at PPP1R11^K59^, but not including MDR2. This indicates that the expression or function of MDR2 is not directly regulated by lactylation, but further research is still needed.

Lactylation of target proteins can not only directly regulate their enzymatic activity but also regulate their protein expression by affecting their structural conformation [51, 52]. For example, LDHA (actate Dehydrogenase A) lactylation at K81/K318 site significantly enhances its enzyme activity rather than its expression, thereby promoting carboplatin resistance in lung adenocarcinoma [53]. Similarity, in myocardial ischemia‐reperfusion injury, K351 lactylation of Serpina3k enhances protein stability, leading to upregulated protein levels and subsequent cardioprotective effects [51]. Interestingly, the lactylation decrease destabilizes PPP1R11 protein and enhances its E3 ligase activity, promoting MDR2 ubiquitination. However, other TME factors may also regulate PPP1R11 through complex networks, as suggested by the finding of Dichtl et al. that Kla may is a consequence rather than a cause of protein expression [22]. However, we could not completely exclude other factors that may also facilitate PPP1R11 upregulation, since TME contain various effectors to affect it expression via the constitution of complicated expression regulatory network. Actually, the activation of Wnt, HIF‐1α, PI3K/AKT, and STAT3 pathways known PPP1R11 regulators, are reported to control PPP1R11 expression [54, 55]. Furthermore, bioinformatic analysis revealed that there were potential POU2F1 binding sites (TAAT core sequence) in the PPP1R11 promoter, suggesting that POU2F1 might regulated the transcriptional expression of PPP1R11. The possibilities the involvement of other mechanisms in our model may explain the results that POU2F1 can not completely diminish the PPP1R11 expression. Notably, we observed spatial proximity between lactylation and ubiquitination sites (e.g., K63), suggesting potential crosstalk in protein stability regulation.

While numerous proteins contain lactylation (Kla) sites, the enzymatic regulation of this modification remains incompletely understood. Current evidence shows that acetyltransferases (e.g., p300) and deacetylases (e.g., HDAC1‐3) can catalyze lactylation and delactylation, respectively [56, 57, 58, 59]. Several lysine acetyltransferases, including TIP60 and KAT2A/ACSS2, have been shown to mediate protein lactylation. For example, it has been reported that TIP60 (also named KAT5, an effective histone acetyltransferase)‐mediated Vps34 lactylation participated in promoting its lipid kinase activity to facilitate cell autophagy and endolysosomal degradationn [60]. Moreover, a new study has pointed that KAT2A, coupled with ACSS2 (acetyl‐CoA synthetase 2), functions as a lactyltransferase and induces lactylation on H3K14 and H3K18 [50]. Herein, POU2F1 was found to reduce PPP1R11 lactylation, thereby stabilizing the protein and maintaining its E3 enzymatic activity. However, the specific lactyltransferase/delactylase regulating PPP1R11 remains unidentified. Interestingly, our proteomic analysis revealed KAT7 upregulation upon POU2F1 silencing. Although KAT7's potential as a lactyltransferase requires validation [61], these findings suggest a possible role in PPP1R11 regulation. Further studies are needed to identify the complete enzymatic machinery controlling PPP1R11 lactylation.

In summary, our study reveals POU2F1 as a novel regulator of MDR2 ubiquitination and degradation, uncovering the POU2F1‐MCT4‐PPP1R11 lactylation axis as a key mechanism controlling MDR2 homeostasis. These findings not only provide new therapeutic targets for overcoming chemoresistance through POU2F1 inhibition, but also offer crucial insights for optimizing targeted intervention strategies against CRC chemoresistance.

## 4 Experimental Section

### 4.1 Data Availability

Data on the POU2F1 expression in colon adenocarcinoma (COAD) were obtained from TCGA (https://gdc.cancer.gov/). RNA‐Seq data reported in this paper were deposited in the NCBI's GEO database (https://www.ncbi.nlm.nih.gov/geo). The human colorectal cancer RNA‐Seq data set and survival data used in Figure S1 was obtained from the GEO database with accession number GSE3964, GSE69657, GSE72968, and TCGA cohort. All patients underwent an assessment of tumor status at baseline and every 6 weeks after chemotherapy by abdominal/pelvic/chest computerized tomography (CT) or magnetic resonance imaging (MRI) and the best response was classified as a complete response (CR), partial response (PR), stable disease (SD), or progressive disease (PD) according to response evaluation criteria in solid tumors (RECIST) standard 1.1 as previously described. Overall survival was defined as the time from the date of chemotherapy to the date of death from any cause or latest follow‐up. The public data from GEO database and TCGA cohort were transformed by log_2_, and analyzed using Excel and GraphPad Prism 9.0 software.

The potential functions of DEPs and DEGs in POU2F1 silencing SW620 cells were analyzed by the GO enrichment analysis. The DEGs in POU2F1 silencing SW620 cells were identified by a fold change of ≥1.5, or ≤ −2.0 and *p*‐value <0.05, determined by Student *t*‐test. The transcriptome profiles of POU2F1 silencing in SW620 cells were analyzed. Hierarchical clustering of DEGs and DEPs was performed using the package R software and expressed by heatmap. Volcano maps were drawn using the ggplot2 packages. All unique/stable reagents generated in this study are available from the corresponding author with a completed material transfer agreement.

### 4.2 Lactylation Proteomics Analysis

POU2F1 silencing SW620 cells were cultured in Dulbecco's Modified Eagle Medium (DMEM) medium supplemented with 10 mM glucose for 24 h before harvest. The collected cells were washed with ice‐cold phosphate‐buffered saline and resuspended in a lysis buffer (8 M urea, pH 7.4, 3 µM TSA, 50 mM NAM, and 1% protease inhibitor cocktail) for sonication on ice. The supernatants were collected after centrifugation at 12 000 g at 4°C for 15 min and the following sequencing was outsourced to Jingjie Biotech that offers high‐sensitivity lactylation sequencing using advanced pan‐antilactyllysine antibody enrichment coupled with high‐resolution mass spectrometry (MS) to profile lysine lactylation (Kla) modifications in proteins.

### 4.3 Plasmids Construction

The molecular cloning of the plasmids used in this study has followed a protocol described previously [14]. The target sequences specific for POU2F1 (1#: 5'‐GCTGTGACGAATCTTTCAGTT‐3' and 2#: 5'‐CCAGTCAACACCAAAGCGAAT‐3'), POU2F1 overexpressing plasmid containing synonymous mutation sequences with two interfering sites, myc‐MDR2 and His‐MCT4 were constructed by GENE Company (Shanghai, China). The plasmids for the expression of HA‐Ub WT and mutant K6, 11 27, 29, 33, 48, and 63 were purchased from GENE (Hong Kong, China). Different mutants of myc‐MDR2 and His‐MCT4 were generated by site‐directed mutagenesis PCR reaction using platinum PWO SuperYield DNA polymerase (Roche, Basel, Switzerland) according to the product manual. PCR reactions. The pcDNA3.1‐Flag‐PPP1R11 vector was constructed by inserting synthesized cDNA encoding 3 × Flag tag and PPP1R11 into pcDNA3.1 vector using EcoRI/XhoI MCS. Mutant K59 plasmids of PPP1R11 were purchased from GENE (Hong Kong, China). All constructs were confirmed by DNA sequencing. HEK293T cells were transfected with individual types of plasmids using Lipofectamine 3000 reagent (Invitrogen, Waltham, MA), according to the manufacturer's instructions.

### 4.4 Antibodies and Reagents

The primary antibodies for POU2F1 (#8157, CST), PPP1R11 (ab171960, Abcam), MDR2 (PA5‐78692, Invitrogen), HA tag (#3724, CST), His tag (#12698, CST), myc tag (ab32, Abcam), Flag tag (MA1‐91878, Invitrogen), MDR1 (#13978, CST), MRP1 (#72202, CST), MRP2 (#4446, CST), LRP (ab27309, Abcam), TOP 1 (#79971, CST), TOP 2A (ab52934, Abcam), TYMS (#9045, CST), α‐tubulin (#2144, CST), Kla (PTM‐1401RM, PTM Bio), SMCT2 (ab262934, Abcam), MCT3 (PA5‐115908, Invitrogen), MCT4 (ab191008, Abcam), Ubiquitin (ab134953, Abcam), and α‐tubulin (ab7291, Abcam) were commercially available. Secondary antibodies used in immunofluorescence assay were: AF488‐anti‐Mouse (Invitrogen), AF594‐anti‐rabbit (Invitrogen), AF488‐anti‐rabbit (Invitrogen), AF594‐anti‐mouse (Invitrogen). Secondary antibodies for Western blots were: anti‐rabbit‐HRP (ab6721, Abcam) and anti‐mouse‐HRP (ab6728, Abcam). Small molecular compounds, such as MG‐132 (S2619, Selleck), Bromosulfalein (Cayman), cycloheximide (C1988, Sigma), L‐lactate (L7022, Merck), Glucose (D9434, Merck) RS6212 (HY‐150753, MCE), Syrosingopine (S9907, Selleck), Rotenone (HY‐B1756, MCE), MSC‐4381 (HY‐132301, MCE), and DAPI (0100‐20, Southern Biotech) were also purchased from the indicated suppliers. Chemotherapeutic drugs such as oxaliplatin (S1224) and irinotecan (S1198) were purchased from Selleck.

### 4.5 E3 Ligase Activity Assay

The E3 Ligase activity of PPP1R11 was detected by E3 Ligase Auto‐Ubiquitylation Assay Kit (ab139469, Abcam) in vitro. First, Flag‐PPP1R11 was purified from *E. coli*. The in vitro ubiquitination reactions were performed with purified Flag‐PPP1R11, purified ubiquitin activating enzyme solution (E1), UbcH5a (E2), ubiquitin, DTT, and 2 mM Mg‐ATP, in a final volume of 50 µL at 37°C for 1 h. The reactions were stopped by adding 50µl 2x SDS‐PAGE gel loading buffer and heated at 95°C for 5 min. The ubiquitinated PPP1R11 proteins were detected by western blot analysis using an anti‐Ubiquitin antibody.

### 4.6 PPP1R11 Protein Purification and Lactylation Assays

For Flag‐tagged PPP1R11 protein expression, *E. coli* BL21(DE3) transformed with the pET28a‐KAT8 plasmids was grown at 37°C to A600 0.6–0.8 and induced by 0.5 mM IPTG at 18°C for 20 h. The cell pellets were harvested and sonicated in Buffer A (25 mM Tris‐HCl, pH 8.0, 300 mM NaCl, 20 mM imidazole). The lysate was centrifuged, and the supernatant was loaded onto a 5 mL Ni‐NTA beads column (Millipore). The column was washed by Buffer A and eluted by Elution buffer A (25 mM Tris‐HCl, pH 8.0, 300 mM NaCl, 500 mM imidazole). Proteins were then exchanged into PBS buffer.

Synthesis of l‐lactyl‐CoA was performed as described previously. In vitro lactylation assays (30 µL) contained purified Flag‐PPP1R11 (30 ng), and unlabeled l‐lactyl‐CoA (20 µM) in reaction buffer (50 mM HEPES [pH7.8], 30 mM KCl, 0.25 mM EDTA, 5.0 mM sodium butyrate, 5.0 mM MgCl2, 2.5 mM DTT). Reactions were incubated at 30°C for 2 h. For each assay, reaction products were resolved by 12.5% SDS‐PAGE and analyzed by immunoblotting with anti‐Kla.

### 4.7 In Vivo Xenograft Tumor Models

Female BALB/c nude mice at 4–6 weeks of age were obtained from Animal Experiment Center of Hunan Cancer Hospital and all animal experiments were carried out in accordance with the principles and procedures outlined by the Joint Ethics Committee of the Affiliated Cancer Hospital of Xiangya School of Medicine, Central South University and Hunan Cancer Hospital in China. For subcutaneous tumorigenicity assay, individual mice were injected subcutaneously with the POU2F1‐overexpressing or vector‐transfected HCT116 cells (1 × 10^6^ cells/mice, *n* = 7/group), when the tumor size reached at 200 mm^3^. The mice were treated by intraperitoneal injection twice a week with either physiological saline or therapeutic drugs (oxaliplatin: 5 mg kg^−1^ and irinotecan: 10 mg kg^−1^). The body weight and tumor size were measured every 3 days. The tumor volume was calculated as 0.5 × (L × W^2^), where L is the longest dimension of the tumor and W is the dimension of the tumor perpendicular to L. Thirty days after injection, the mice were sacrificed and the tumors were excised for analysis. In the nude mice survival model, 5 × 10^4^ POU2F1‐overexpressing or vector‐transfected HCT116 cells were subcutaneously injected into mice (*n* = 10/group). Once tumors were established, the mice were treated by intraperitoneal injection twice a week with either physiological saline or therapeutic drugs (oxaliplatin: 5 mg kg^−1^ and irinotecan: 10 mg kg^−1^).  The body weight and physical condition were evaluated every 5 days. The mice were euthanized once the body weight had rapidly decreased to <15 g or their physical condition reached the humane endpoint, and the tumor were harvested for further analysis.

### 4.8 Statistical Analysis

Data are presented as the mean ± standard deviation (SD). The difference between groups was analyzed by student's *t*‐test. The association between the levels of POU2F1 PPP1R11 and MDR2 expression, and the values of clinicopathological parameters was analyzed by the Chi‐square test. The relationship between the levels of POU2F1 and MDR2 or PPP1R11 expression was analyzed by Spearman's rank test. Survival was estimated by the Kaplan–Meier method and compared by log‐rank test. The potential risks of individual factors for the survival of colon cancer patients were analyzed by univariate and multivariate analyses using Cox regression model after adjusting for baseline characteristics. All statistical analyses were performed using the SPSS version 22.0 (SPSS, Chicago, IL). A *p*‐value of <0.05 was considered statistically significant.

### 4.9 Ethical Statement

The use of human tissue and experimental animals in this study was approved by the Joint Ethics Committee of the Affiliated Cancer Hospital of Xiangya School of Medicine, Central South University, and Hunan Cancer Hospital in China. Informed consent was obtained from all participants.

## Author Contributions

Q.L. and Y.Z. conceived and designed all experiments. L.X. and J.L. performed experiments, collected and analyzed the data, and wrote the manuscript. X.J. and Q.P. performed experiments and wrote the manuscript. L.O. and S.T. collected and analyzed the data. L.X., L.O., and S.T. performed experiments. Y.Z. and J.L. provided the clinical samples and reagents. All authors critically reviewed and approved the final manuscript.

## Conflicts of Interest

The authors declare no conflicts of interest.

## Supporting information




**Supporting File**: advs74723‐sup‐0001‐SuppMat.docx.

## Data Availability

The data that support the findings of this study are available in the supplementary material of this article.

## References

[1] R. L. Siegel , T. B. Kratzer , A. N. Giaquinto , H. Sung , and A. Jemal , “Cancer Statistics, 2025,” CA: A Cancer Journal for Clinicians 75, no. 1 (2025): 10–45, 10.3322/caac.21871. 39817679 PMC11745215

[2] T. Andre , K. K. Shiu , T. W. Kim , et al., “Pembrolizumab Versus Chemotherapy in Microsatellite Instability‐High or Mismatch Repair‐Deficient Metastatic Colorectal Cancer: 5‐Year Follow‐Up From the Randomized Phase III KEYNOTE‐177 Study,” Annals of Oncology 36, no. 3 (2025): 277–284, 10.1016/j.annonc.2024.11.012. 39631622

[3] A. Moorman , E. K. Benitez , F. Cambulli , et al., “Progressive Plasticity During Colorectal Cancer Metastasis,” Nature 637, no. 8047 (2025): 947–954, 10.1038/s41586-024-08150-0. 39478232 PMC11754107

[4] M. H. Dias , A. Friskes , S. Wang , et al., “Paradoxical Activation of Oncogenic Signaling as a Cancer Treatment Strategy,” Cancer Discovery 14, no. 7 (2024): 1276–1301, 10.1158/2159-8290.CD-23-0216. 38533987 PMC11215412

[5] J. Deng , T. Pan , D. Wang , et al., “The MondoA‐Dependent TXNIP/GDF15 Axis Predicts Oxaliplatin Response in Colorectal Adenocarcinomas,” EMBO Molecular Medicine 16, no. 9 (2024): 2080–2108, 10.1038/s44321-024-00105-2. 39103698 PMC11393413

[6] D. Dong , X. Yu , J. Xu , N. Yu , Z. Liu , and Y. Sun , “Cellular and Molecular Mechanisms of Gastrointestinal Cancer Liver Metastases and Drug Resistance,” Drug Resistance Updates 77 (2024): 101125, 10.1016/j.drup.2024.101125. 39173439

[7] R. W. Robey , K. M. Pluchino , M. D. Hall , A. T. Fojo , S. E. Bates , and M. M. Gottesman , “Revisiting the Role of ABC Transporters in Multidrug‐Resistant Cancer,” Nature Reviews Cancer 18, no. 7 (2018): 452–464, 10.1038/s41568-018-0005-8. 29643473 PMC6622180

[8] Y. Liu , K. Chen , F. Li , et al., “Probiotic Lactobacillus Rhamnosus GG Prevents Liver Fibrosis Through Inhibiting Hepatic Bile Acid Synthesis and Enhancing Bile Acid Excretion in Mice,” Hepatology 71, no. 6 (2020): 2050–2066, 10.1002/hep.30975. 31571251 PMC7317518

[9] X. Sun , Y. Chen , C. Yang , et al., “Chemical Recording of Pump‐Specific Drug Efflux in Living Cells,” Angewandte Chemie International Edition 63, no. 49 (2024): 202409282, 10.1002/anie.202409282. 39324755

[10] P. Borst and A. H. Schinkel , “P‐Glycoprotein ABCB1: A Major Player in Drug Handling by Mammals,” Journal of Clinical Investigation 123, no. 10 (2013): 4131–4133, 10.1172/JCI70430. 24084745 PMC3784548

[11] A. Cort , T. Ozben , L. Saso , C. De Luca , and L. Korkina , “Redox Control of Multidrug Resistance and Its Possible Modulation by Antioxidants,” Oxidative Medicine and Cellular Longevity 2016 (2016): 4251912, 10.1155/2016/4251912. 26881027 PMC4736404

[12] P. Borst , N. Zelcer , and A. van Helvoort , “ABC Transporters in Lipid Transport,” Biochimica et Biophysica Acta (BBA)—Molecular and Cell Biology of Lipids 1486, no. 1 (2000): 128–144, 10.1016/s1388-1981(00)00053-6. 10856718

[13] R. Ganapathi , T. Kuo , L. Teeter , D. Grabowski , and J. Ford , “Relationship Between Expression of P‐glycoprotein and Efficacy of Trifluoperazine in Multidrug‐Resistant Cells,” Molecular Pharmacology 39, no. 1 (1991): 1–8, 10.1016/S0026-895X(25)10867-5. 1670962

[14] J. Lin , L. Xia , L. Oyang , et al., “The POU2F1‐ALDOA Axis Promotes the Proliferation and Chemoresistance of Colon Cancer Cells by Enhancing Glycolysis and the Pentose Phosphate Pathway Activity,” Oncogene 41, no. 7 (2022): 1024–1039, 10.1038/s41388-021-02148-y. 34997215 PMC8837540

[15] J. Deng , Y. Li , L. Yin , et al., “Histone Lactylation Enhances GCLC Expression and thus Promotes Chemoresistance of Colorectal Cancer Stem Cells Through Inhibiting Ferroptosis,” Cell Death & Disease 16, no. 1 (2025): 193, 10.1038/s41419-025-07498-z. 40113760 PMC11926133

[16] A. De Leo , A. Ugolini , X. Yu , et al., “Glucose‐Driven Histone Lactylation Promotes the Immunosuppressive Activity of Monocyte‐Derived Macrophages in Glioblastoma,” Immunity 57, no. 5 (2024): 1105–1123.e8, 10.1016/j.immuni.2024.04.006. 38703775 PMC11114377

[17] D. Zhang , Z. Tang , H. Huang , et al., “Metabolic Regulation of Gene Expression by Histone Lactylation,” Nature 574, no. 7779 (2019): 575–580, 10.1038/s41586-019-1678-1. 31645732 PMC6818755

[18] Y. Chen , J. Wu , L. Zhai , et al., “Metabolic Regulation of Homologous Recombination Repair by MRE11 Lactylation,” Cell 187, no. 2 (2024): 294–311.e21, 10.1016/j.cell.2023.11.022. 38128537 PMC11725302

[19] K. Sato , A. Mizutani , Y. Muranaka , et al., “Biological Distribution After Oral Administration of Radioiodine‐Labeled Acetaminophen to Estimate Gastrointestinal Absorption Function via OATPs, OATs, and/or MRPs,” Pharmaceutics 15, no. 2 (2023): 497, 10.3390/pharmaceutics15020497. 36839818 PMC9964641

[20] A. C. McKelvey , T. B. Lear , S. R. Dunn , et al., “RING Finger E3 Ligase PPP1R11 Regulates TLR2 Signaling and Innate Immunity,” Elife 5 (2016), e18496, 10.7554/eLife.18496. 27805901 PMC5092053

[21] W. Liu , Y. Wang , L. H. M. Bozi , et al., “Lactate Regulates Cell Cycle by Remodelling the Anaphase Promoting Complex,” Nature 616, no. 7958 (2023): 790–797, 10.1038/s41586-023-05939-3. 36921622 PMC12175651

[22] S. Shu , T. Liu , X. Yang , et al., “Alpha‐enolase influences ATP pool of cytoplasm and lactate homeostasis by regulating glycolysis in gastric cancer,” Signal Transduction and Targeted Therapy 10, no. 1 (2025), 356, 10.1126/sciadv.abg3505. 41168198 PMC12575808

[23] Z. Yang , C. Yan , J. Ma , et al., “Lactylome Analysis Suggests Lactylation‐Dependent Mechanisms of Metabolic Adaptation in Hepatocellular Carcinoma,” Nature Metabolism 5, no. 1 (2023): 61–79, 10.1038/s42255-022-00710-w. 36593272

[24] J. Chen , D. Zhao , L. Zhang , et al., “Tumor‐Associated Macrophage (TAM)‐Secreted CCL22 Confers Cisplatin Resistance of Esophageal Squamous Cell Carcinoma (ESCC) Cells Via Regulating the Activity of Diacylglycerol Kinase α (DGKα)/NOX4 Axis,” Drug Resistance Updates 73 (2024): 101055, 10.1016/j.drup.2024.101055. 38387281

[25] S. Barzegar and S. Pirouzpanah , “Zinc Finger Proteins and ATP‐Binding Cassette Transporter‐Dependent Multidrug Resistance,” European Journal of Clinical Investigation 54, no. 2 (2024): 14120, 10.1111/eci.14120. 37930002

[26] C. Wen , L. Fu , J. Huang , et al., “Curcumin Reverses Doxorubicin Resistance Via Inhibition the Efflux Function of ABCB4 in Doxorubicin‑Resistant Breast Cancer Cells,” Molecular Medicine Reports 19, no. 6 (2019): 5162–5168, 10.3892/mmr.2019.10180. 31059026 PMC6522915

[27] H. Hu , M. Wang , X. Guan , et al., “Loss of ABCB4 Attenuates the Caspase‐Dependent Apoptosis Regulating Resistance to 5‐Fu in Colorectal Cancer,” Bioscience Reports 38, no. 1 (2018), BSR20171428, 10.1042/BSR20171428. 29371412 PMC5821943

[28] M. Awoniyi , J. Wang , B. Ngo , et al., “Protective and Aggressive Bacterial Subsets and Metabolites Modify Hepatobiliary Inflammation and Fibrosis in a Murine Model of PSC,” Gut 72, no. 4 (2023): 671–685, 10.1136/gutjnl-2021-326500. 35705368 PMC9751228

[29] A. Shriki , T. Lanton , A. Sonnenblick , et al., “Multiple Roles of IL6 in Hepatic Injury, Steatosis, and Senescence Aggregate to Suppress Tumorigenesis,” Cancer Research 81, no. 18 (2021): 4766–4777, 10.1158/0008-5472.CAN-21-0321. 34117031

[30] D. Coe , T. Poobalasingam , H. Fu , et al., “Loss of Voltage‐Gated Hydrogen Channel 1 Expression Reveals Heterogeneous Metabolic Adaptation to Intracellular Acidification by T Cells,” JCI Insight 7, no. 10 (2022), e147814, 10.1172/jci.insight.147814. 35472029 PMC9220931

[31] P. Wu , Y. Zhou , Y. Guo , S. L. Zhang , and K. Y. Tam , “Recent Developments of Human Monocarboxylate Transporter (hMCT) Inhibitors as Anticancer Agents,” Drug Discovery Today 26, no. 3 (2021): 836–844, 10.1016/j.drudis.2021.01.003. 33450176

[32] I. Dell'Anno , E. Barone , L. Mutti , et al., “Tissue Expression of Lactate Transporters (MCT1 and MCT4) and Prognosis of Malignant Pleural Mesothelioma (Brief Report),” Journal of Translational Medicine 18, no. 1 (2020): 341, 10.1186/s12967-020-02487-6. 32887638 PMC7650278

[33] V. L. Payen , E. Mina , V. F. Van Hee , P. E. Porporato , and P. Sonveaux , “Monocarboxylate Transporters in Cancer,” Molecular Metabolism 33 (2020): 48–66, 10.1016/j.molmet.2019.07.006. 31395464 PMC7056923

[34] X. Li , S. Geng , Q. Chen , et al., “Disrupting Tumor Lactate Homeostasis to Sensitize Chemo‐Immunotherapy Using a Glucose‐Disguised Lactate Interceptor,” ACS Nano 19, no. 23 (2025): 21556–21570, 10.1021/acsnano.5c03545. 40472333

[35] Y. Sun , H. Wang , Z. Cui , et al., “Lactylation in Cancer Progression and Drug Resistance,” Drug Resistance Updates 81 (2025): 101248, 10.1016/j.drup.2025.101248. 40287994

[36] J. Dai , X. Lu , C. Zhang , et al., “NNMT Promotes Acquired EGFR‐TKI Resistance by Forming EGR1 and Lactate‐Mediated Double Positive Feedback Loops in Non‐Small Cell Lung Cancer,” Molecular Cancer 24, no. 1 (2025): 79, 10.1186/s12943-025-02285-y. 40089784 PMC11909984

[37] K. Sun , Y. Shi , C. Yan , et al., “Glycolysis‐Derived Lactate Induces ACSL4 Expression and Lactylation to Activate Ferroptosis During Intervertebral Disc Degeneration,” Advanced Science 12 (2025): 2416149, 10.1002/advs.202416149. 40171826 PMC12140309

[38] W. D. Lee , D. R. Weilandt , L. Liang , et al., “Lactate Homeostasis is Maintained Through Regulation of Glycolysis and Lipolysis,” Cell Metabolism 37, no. 3 (2025): 758–771.e8, 10.1016/j.cmet.2024.12.009. 39889702 PMC11926601

[39] K. Sun , X. Zhang , J. Shi , et al., “Mature T Cell Responses are Controlled by MicroRNA‐142,” Journal of Clinical Investigation 125, no. 7 (2025): 2825–2840, 10.1172/JCI187024. PMC4563679 26098216

[40] L. Wang , S. Bi , Z. Li , et al., “Napabucasin Deactivates STAT3 and Promotes Mitoxantrone‐Mediated cGAS‐STING Activation for Hepatocellular Carcinoma Chemo‐Immunotherapy,” Biomaterials 313 (2025): 122766, 10.1016/j.biomaterials.2024.122766. 39180916

[41] M. P. Plebanek , Y. Xue , Y. V. Nguyen , et al., “A Lactate‐SREBP2 Signaling Axis Drives Tolerogenic Dendritic Cell Maturation and Promotes Cancer Progression,” Science Immunology 9, no. 95 (2024): adi4191, 10.1126/sciimmunol.adi4191. PMC11926670 38728412

[42] Y. Zeng , Y. Huang , Q. Tan , et al., “Influence of Lactate in Resistance to Anti‑PD‑1/PD‑L1 Therapy: Mechanisms and Clinical Applications (Review),” Molecular Medicine Reports 31, no. 2 (2025), 48, 10.3892/mmr.2024.13413. 39670310 PMC11650113

[43] J. Cai , L. Song , F. Zhang , et al., “Targeting SRSF10 Might Inhibit M2 Macrophage Polarization and Potentiate Anti‐PD‐1 Therapy in Hepatocellular Carcinoma,” Cancer Communications 44, no. 11 (2024): 1231–1260, 10.1002/cac2.12607. 39223929 PMC11570766

[44] Y. He , T. Song , J. Ning , et al., “Lactylation in Cancer: Mechanisms in Tumour Biology and Therapeutic Potentials,” Clinical and Translational Medicine 14, no. 11 (2024): 70070, 10.1002/ctm2.70070. PMC11511673 39456119

[45] G. Li , D. Wang , Y. Zhai , et al., “Glycometabolic Reprogramming‐Induced XRCC1 Lactylation Confers Therapeutic Resistance in ALDH1A3‐Overexpressing Glioblastoma,” Cell Metabolism 36, no. 8 (2024): 1696–1710.e10, 10.1016/j.cmet.2024.07.011. 39111285

[46] A. N. Chen , Y. Luo , Y. H. Yang , et al., “Lactylation, a Novel Metabolic Reprogramming Code: Current Status and Prospects,” Frontiers in Immunology 12 (2021): 688910, 10.3389/fimmu.2021.688910. 34177945 PMC8222712

[47] H. Chen , Y. Li , H. Li , et al., “NBS1 Lactylation is Required for Efficient DNA Repair and Chemotherapy Resistance,” Nature 631, no. 8021 (2024): 663–669, 10.1038/s41586-024-07620-9. 38961290 PMC11254748

[48] F. Li , H. Zhang , Y. Huang , et al., “Single‐cell Transcriptome Analysis Reveals the Association Between Histone Lactylation and Cisplatin Resistance in Bladder Cancer,” Drug Resistance Updates 73 (2024): 101059, 10.1016/j.drup.2024.101059. 38295753

[49] H. Hong , X. Chen , H. Wang , X. Gu , Y. Yuan , and Z. Zhang , “Global Profiling of Protein Lysine Lactylation and Potential Target Modified Protein Analysis in Hepatocellular Carcinoma,” Proteomics 23, no. 9 (2023): 2200432, 10.1002/pmic.202200432. 36625413

[50] R. Zhu , X. Ye , X. Lu , et al., “ACSS2 Acts as a Lactyl‐Coa Synthetase and Couples KAT2A to Function as a Lactyltransferase for Histone Lactylation and Tumor Immune Evasion,” Cell Metabolism 37, no. 2 (2025): 361–376.e7, 10.1016/j.cmet.2024.10.015. 39561764

[51] L. Wang , D. Li , F. Yao , et al., “Serpina3k Lactylation Protects from Cardiac Ischemia Reperfusion Injury,” Nature Communications 16, no. 1 (2025): 1012, 10.1038/s41467-024-55589-w. PMC11760901 39856050

[52] H. Li , C. Liu , R. Li , et al., “AARS1 and AARS2 Sense L‐Lactate to Regulate cGAS as Global Lysine Lactyltransferases,” Nature 634, no. 8036 (2024): 1229–1237, 10.1038/s41586-024-07992-y. 39322678

[53] J. Li , W. Xun , X. Wang , et al., “Lactylation Enhances the Activity of Lactate Dehydrogenase A and Promotes the Chemoresistance to Cisplatin Through Facilitating DNA Nonhomologous End Junction in Lung Adenocarcinoma,” Advanced Science 13, no. 3 (2026): 10733, 10.1002/advs.202510733. PMC12806394 41190808

[54] C. Seidl , F. D. Silva , K. Zhang , K. Wohlgemuth , H. Omran , and C. Niehrs , “Mucociliary Wnt Signaling Promotes Cilia Biogenesis and Beating,” Nature Communications 14, no. 1 (2023): 1259, 10.1038/s41467-023-36743-2. PMC9988884 36878953

[55] H. Li , M. Rokavec , L. Jiang , D. Horst , and H. Hermeking , “Antagonistic Effects of p53 and HIF1A on MicroRNA‐34a Regulation of PPP1R11 and STAT3 and Hypoxia‐Induced Epithelial to Mesenchymal Transition in Colorectal Cancer Cells,” Gastroenterology 153, no. 2 (2017): 505–520, 10.1053/j.gastro.2017.04.017. 28435028

[56] B. Xie , M. Zhang , J. Li , et al., “KAT8‐Catalyzed Lactylation Promotes eEF1A2‐Mediated Protein Synthesis and Colorectal Carcinogenesis,” Proceedings of the National Academy of Sciences USA 121, no. 8 (2024): 2314128121, 10.1073/pnas.2314128121. PMC10895275 38359291

[57] A. Ziogas , B. Novakovic , L. Ventriglia , et al., “Long‐ Term Histone Lactylation Connects Metabolic and Epigenetic Rewiring in Innate Immune Memory,” Cell 188, no. 11 (2025): 2992–3012.e16, 10.1016/j.cell.2025.03.048. 40318634

[58] Z. Zong , J. Ren , B. Yang , L. Zhang , and F. Zhou , “Emerging Roles of Lysine Lactyltransferases and Lactylation,” Nature Cell Biology 27, no. 4 (2025): 563–574, 10.1038/s41556-025-01635-8. 40185947

[59] C. Moreno‐Yruela , D. Zhang , W. Wei , et al., “Class I Histone Deacetylases (HDAC1–3) are Histone Lysine Delactylases,” Science Advances 8, no. 3 (2022): abi6696, 10.1126/sciadv.abi6696. PMC8769552 35044827

[60] M. Jia , X. Yue , W. Sun , et al., “ULK1‐ Mediated Metabolic Reprogramming Regulates Vps34 Lipid Kinase Activity By its Lactylation,” Science Advances 9, no. 22 (2023): adg4993, 10.1126/sciadv.adg4993. PMC10413652 37267363

[61] L. Liu , J. Zhang , Z. Dong , et al., “Histone Lactylation–Mediated Metabolic Remodeling in Vascular Smooth Muscle Cells Aggravates Aortic Aneurysm and Dissection by Promoting Lactate Accumulation,” Circulation 153, no. 3 (2026): 189–209, 10.1161/CIRCULATIONAHA.125.072576. 41487086 PMC12952488

