# Correction to “POU2F1 Promotes Chemoresistance in Colorectal Cancer Cells via Attenuates the MDR2 Degradation Mediated by PPP1R11 Lactylation”

**DOI:** 10.1002/advs.76815

**Published:** 2026-07-30

L. Xia, J. Lin, X. Jiang, et al. “POU2F1 Promotes Chemoresistance in Colorectal Cancer Cells via Attenuates the MDR2 Degradation Mediated by PPP1R11 Lactylation,” *Advanced Science* 13 no. 28 (2026): e22316, https://doi.org/10.1002/advs.202522316.

In section 2.5 of the Results, the authors found that Figure 6 is identical to supplementary Figure S6, which does not match the results described and the corresponding figure legend. After cross‐checking the submission history, the authors found that the correct Figure 6 was originally uploaded in the initial submission, but during the revision submission, Supplementary Figure S6 was mistakenly uploaded as Figure 6, and this error was not caught in time during proofreading. The correct Figure 6 is presented below.

**Figure:**
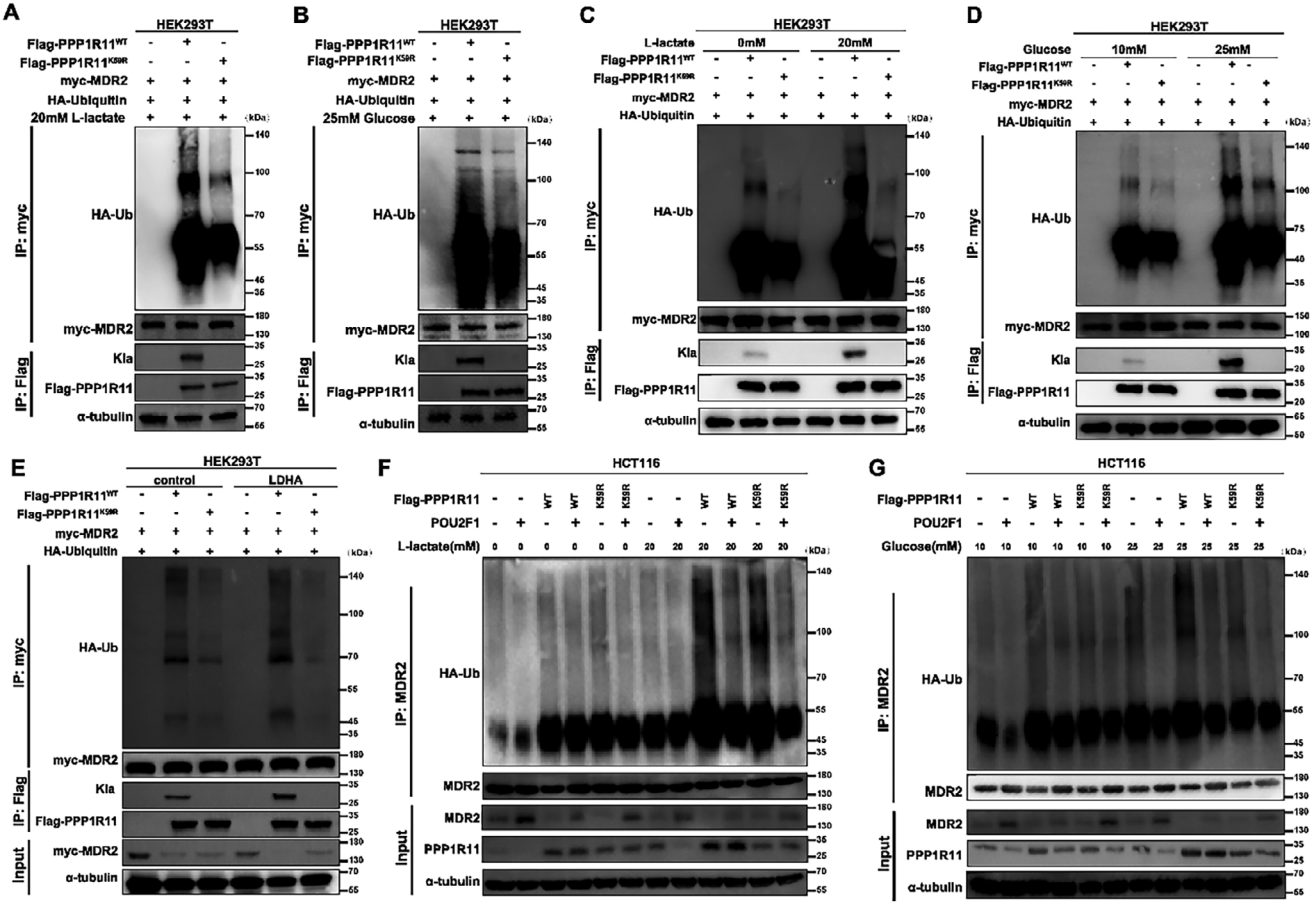
Figure 6 POU2F1 alleviates PPP1R11‐mediated MDR2 ubiquitination via restraining PPP1R11 lactylation. (A–D) Immunoblots for PPP1R11 lactylation and MDR2 ubiquitination in HEK293T cells expressing a vector control, Flag‐tagged PPP1R11, Flag‐tagged PPP1R11‐K59R, myc‐MDR2, and HA‐ubiquitin in the absence or presence of 20 mM lactate (A, C) or 10 mM/25 mM glucose (B, D) for 24 h. (E) Western blot analyses for PPP1R11 lactylation and MDR2 ubiquitination in HEK293T si‐LDHA cells expressing a vector control, Flag‐tagged PPP1R11, Flag‐tagged PPP1R11‐K59R, myc‐MDR2, and HA‐ubiquitin in the presence of 10 mM glucose for 24 h. (F, G) Immunoblots for PPP1R11 lactylation and MDR2 ubiquitination in POU2F1‐overexpressing HCT116 cells expressing a vector control, Flag‐tagged PPP1R11, Flag‐tagged PPP1R11‐K59R, myc‐MDR2, and HA‐ubiquitin in the absence or presence of 20 mM lactate (F) or 10 mM/25 mM glucose (G) for 24 h.

The authors sincerely apologize for this error.

