# Supplementary material for: POU2F1 Promotes Chemoresistance in Colorectal Cancer Cells via Attenuates the MDR2 Degradation Mediated by PPP1R11 Lactylation
Source: Adv Sci (Weinh). 2026 Mar 7;13(28):e22316. doi: 10.1002/advs.202522316 (PMC13185832; doi:10.1002/advs.202522316)
Supplement: Supplementary file 1 — Supporting File: advs74723‐sup‐0001‐SuppMat.docx. [file ADVS-13-e22316-s001.docx]

**Supplementary materials**

**Cell Culture**

Human colon cancer cell lines, HCT116, SW620, and HEK293T cells were obtained from the Cancer Research Institute of Central South University with STR identification, HCT116/L and SW620/Ir cells were constructed by our laboratory without STR identification, respectively. Cells were routinely tested for mycoplasma contamination (MP0035, Sigma-Aldrich), and only negative cells were used in experiments. The SW620, HCT116, HCT116/L, SW620/Ir, POU2F1 silencing SW620, and POU2F1 overexpressing HCT116 cells were cultured in RPMI-1640 (Gibco, USA), and the HEK293T cells in DMEM (Gibco) supplemented with 10% FBS (Zeta Life, France), 100 Units/ml penicillin, and 100 μg/ml streptomycin (Gibco) at 37°C in 5% CO_2_.

**Cell Proliferation and Clonogenic survival assay**

Cell viability was determined using the Cell Counting Kit‐8 (CCK‐8; Dojindo, Japan) according to the manufacturer's instructions. Briefly, the cells (5000 cells/well) were incubated with CCK‐8 reagent for 2h at 37°C at different time points as indicated, and the absorbance at 450nm was measured using a microplate reader (Bio‐Rad Laboratories, Inc.). To determine clonogenicity of individual groups of cells, the cells (2000 cells/dish) were cultured in 6-cm dishes and exposed to different fixed-ratios of oxaliplatin and irinotecan for 10-14 days, fixed with methanol and stained with 0.1% crystal violet. The visible colonies were counted in a blinded manner.

***In vitro* ubiquitination assays**

Both *in vivo* and *in vitro* ubiquitination assays were performed as previous report [39]. Brieﬂy, HEK293T cells were transfected with the plasmids for the expression of POU2F1, Flag-PPP1R11, myc-MDR2 and HA-ubiquitin for two days. Their cell lysates were prepared and boiled in 1% SDS RIPA buffer for 5 min. The same number of proteins from each group was immunoprecipitated with anti-HA, anti-Flag or anti-myc microbeads and after being washed, the bound proteins were resolved by Western blot using anti-HA, anti-Flag and anti-myc to view MDR2 or PPP1R11 ubiquitination.

**Quantification of extracellular/ cytosolic lactate**

Lactate levels were measured by using a colorimetric lactate assay according to the manufacturer’s protocol (Solarbio, YZ-K-LATE).

**Dual luciferase assay and Chromatin immunoprecipitation (ChIP) assay**

The activity of the -1900 to +50 regions of the MCT4 promoter was tested by dual luciferase assays. Individual groups of cells were co-transfected with the plasmids for one fragment in the region to control luciferase expression, together with the Renilla luciferase reporter for 24 h. The levels of firefly and Renilla luciferase activities were measured using a Dual-Luciferase Reporter Assay System (Promega). The binding of POU2F1 to the MCT4 promoter was analyzed by ChIP using a Chromatin Immunoprecipitation Assay Kit (EMD Millipore), according to the manufacturer’s instruction. Briefly, individual groups of cells (1×10^7^/tube) were harvested and their genomic DNA was extracted and reacted with anti-POU2F1 or control IgG. After IP, the contained DNA was analyzed by PCR using the primers. The sequences of primers were showed in **Tables S1**. The PCR products were resolved on 2% agarose gels.

**RNA extraction and quantitative real-time PCR (qRT-PCR)**

Total cellular RNA was extracted using TRIzol reagent (Invitrogen, 15596-018), and reversely transcribed into cDNA using Revert Aid First Strand cDNA Synthesis Kit (Thermo scientific, Massachusetts, USA), according to the manufacturer’s instruction. The relative levels of targeted gene mRNA transcripts to the α-tubulin were determined by qRT-PCR using a Fast Start Essential DNA Green Master kit (Lifescience, Roche, Mannheim, Germany) and specific primers (**Table S2**) in the Roche Light Cycler® 96 Instrument (Lifescience). The PCR reactions were performed in triplicate at 95°C for 5 min and subjected to 40 cycles of 95°C (30 s), 60°C (10 s), and 72°C (1 min). The data were analyzed by 2^−∆∆Ct^.

**Immunohistochemistry**

Immunohistochemistry (IHC) analysis using paraffin embedded CRC specimens was conducted following standard manufacturer’s protocols as described previously. Primary antibodies anti-POU2F1 (#8157, CST), anti-PPP1R11 (ab171960, Abcam), anti-MDR2 (Invitrogen) were used for IHC staining. IHC staining was evaluated by two independent gastrointestinal pathologists blinded to the patients’ characteristics and clinical outcomes. Final IHC score was calculated based on both the extent and the intensity of staining. The staining extent that scored according to the percentage of positively stained cells ranged from 0 to 3 (0, 0–5%; 1, 5–25%; 2, 26–50%; 3, 51–75%; and 4, 76–100%), although the intensity of staining was scored as 0 (negative staining), 1 (weak staining), 2 (moderate staining), and 3 (strong staining). The Cutoff Finder program was used to determine the optimal cutoff for POU2F1, PPP1R11, and MDR2expression. Specimens with the final scores ≥ 3 were defined as high expression, and specimens with the final scores < 3 were defined as low expression.

**Immunofluorescent staining**

The paraffin embedded CRC specimens were fixed with methanol and then gently washed with PBS (Biosharp, BL-601A) twice. Then, the cells were incubated with permeabilization buffer (eBioscience, 00-8333-56) for 10 min at 4°C. After blocking in 5% BSA in TBS/Tween-20 for 1 h at room temperature, the cells were incubated with primary antibodies against POU2F1 and MDR2 at 4°C overnight. The secondary antibody was marked with fluorescence in the dark, and DAPI was applied. The images were captured using a confocal microscopy (LSM510 META, ZEISS, Germany).

**Western blot**

Post-treatment, cells were washed twice with cold PBS before lysis with RIPA buffer (50 mM Tris–HCl pH 7.5, 150 mM NaCl, 0.1% sodium deoxycholate, 0.1% SDS, 1 mM EDTA pH 8.0, 1% NP-40) containing a proteinase and phosphatase inhibitor cocktail (Thermo Fisher). After incubation in lysis buffer for 30 min on ice, cells lysates were collected by using a cell-scraper and centrifuged for 15 min at 10,000 r.p.m. at 4 °C, and the supernatant was collected. Individual cell lysates (30μg) were separated by sodium dodecyl sulfate-polyacrylamide gel electrophoresis (SDS-PAGE) on 10% gels, and transferred onto PVDF membranes. After being blocked with 5% fat-free dry milk in TBST, the membranes were incubated overnight at 4°C with primary antibodies (**Tables S3**). The bound antibodies were detected by horseradish peroxidase-conjugated second antibodies, and visualized using Pierce™ ECL Western Blotting Substrate (Thermo Scientific). The levels of targeting proteins were quantified densitometric scanning using ImageJ software.

**Figure legends**

**
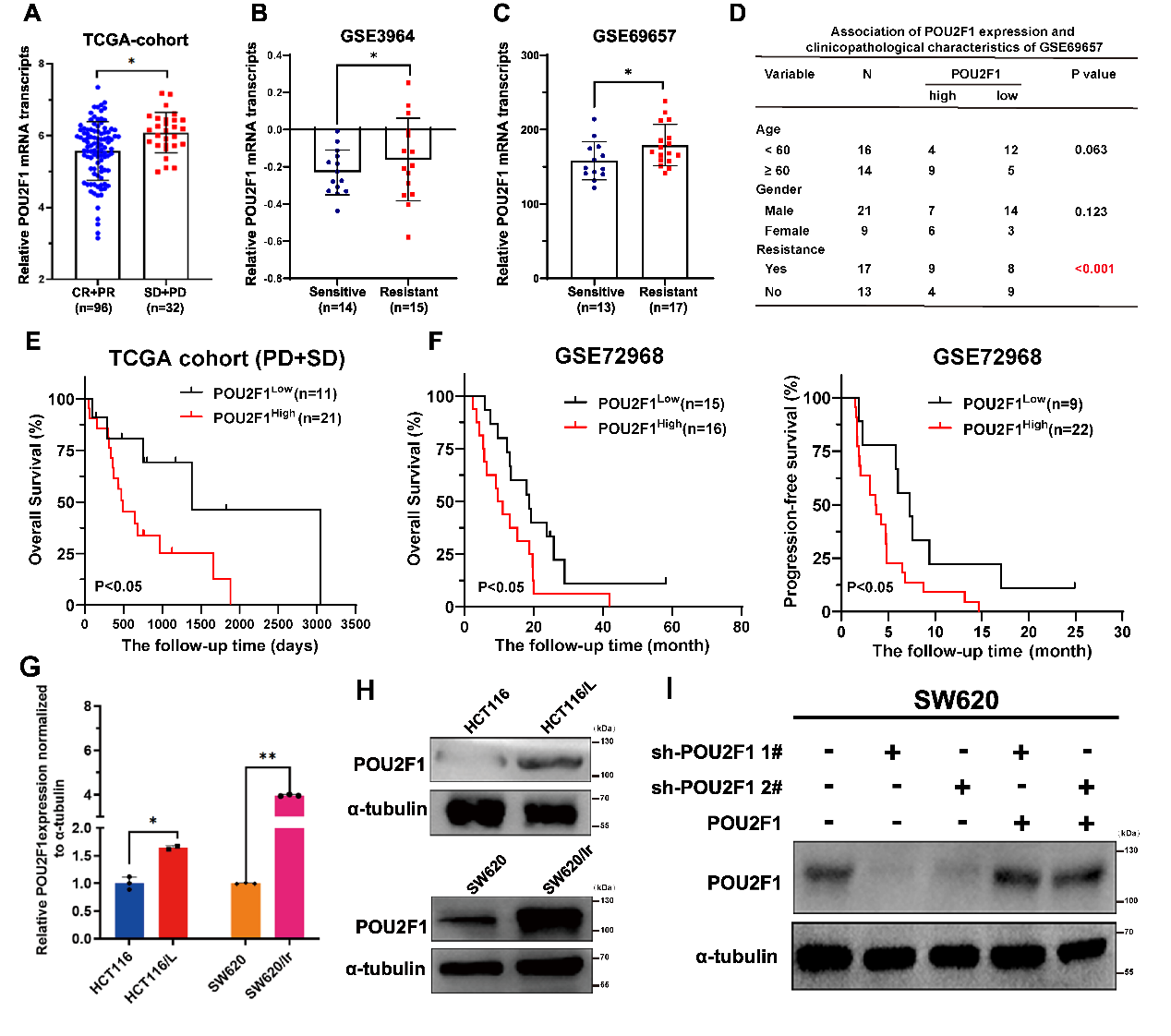
**

**Figure S1 POU2F1 is highly expressed in chemoresistant CRC tissues.**

(**A**) Scatter plot displayed the levels of POU2F1 mRNA transcripts in COAD patients with partial response/complete response (PR/CR, responders, n=96) compared with patients with progressive disease/stable disease (PD/SD, non-responders, n=32) after primary therapy based on TCGA cohort. (**B**, **C**) Plot chart comparing the expression of POU2F1 in sensitive CRC tissues compared with resistant tissues in GSE3964 (sensitive: n=14, resistant: n=15, **B**) and GSE69657 (sensitive: n=13, resistant: n=17, **C**) dataset, respectively. (**D**) Association between POU2F1 mRNA transcripts and clinicopathological characteristics of CRC patients deprived from GSE69657 dataset. (**E**) Kaplan-Meier analysis of OS in colorectal cancer patients with progressive disease/stable disease (PD/SD, non-responders) after stratification with the median value of POU2F1 mRNA transcripts in TCGA. (**F**) Kaplan-Meier analysis of OS and PFS in chemoresistant colorectal cancer patients after stratification with the median value of POU2F1 mRNA transcripts in colorectal cancer patients with progressive disease/stable disease (PD/SD, non-responders) derived from GSE72968 dataset. (**G**, **H**) RT-qPCR and Western blotting analyses of the relative levels of POU2F1 expression in oxaliplatin-resistant and control HCT116 cells, irinotecan-resistant and control SW620 cells, respectively. (**I**) Western blot analysis of the relative levels of POU2F1 expression in the indicated groups of SW620 cells. Data are representative images or expressed as the mean ± SD of each group of samples analyzed in triplicate from three separate experiments. *P<0.05, **P<0.01, ***P<0.001, ns, no significance.


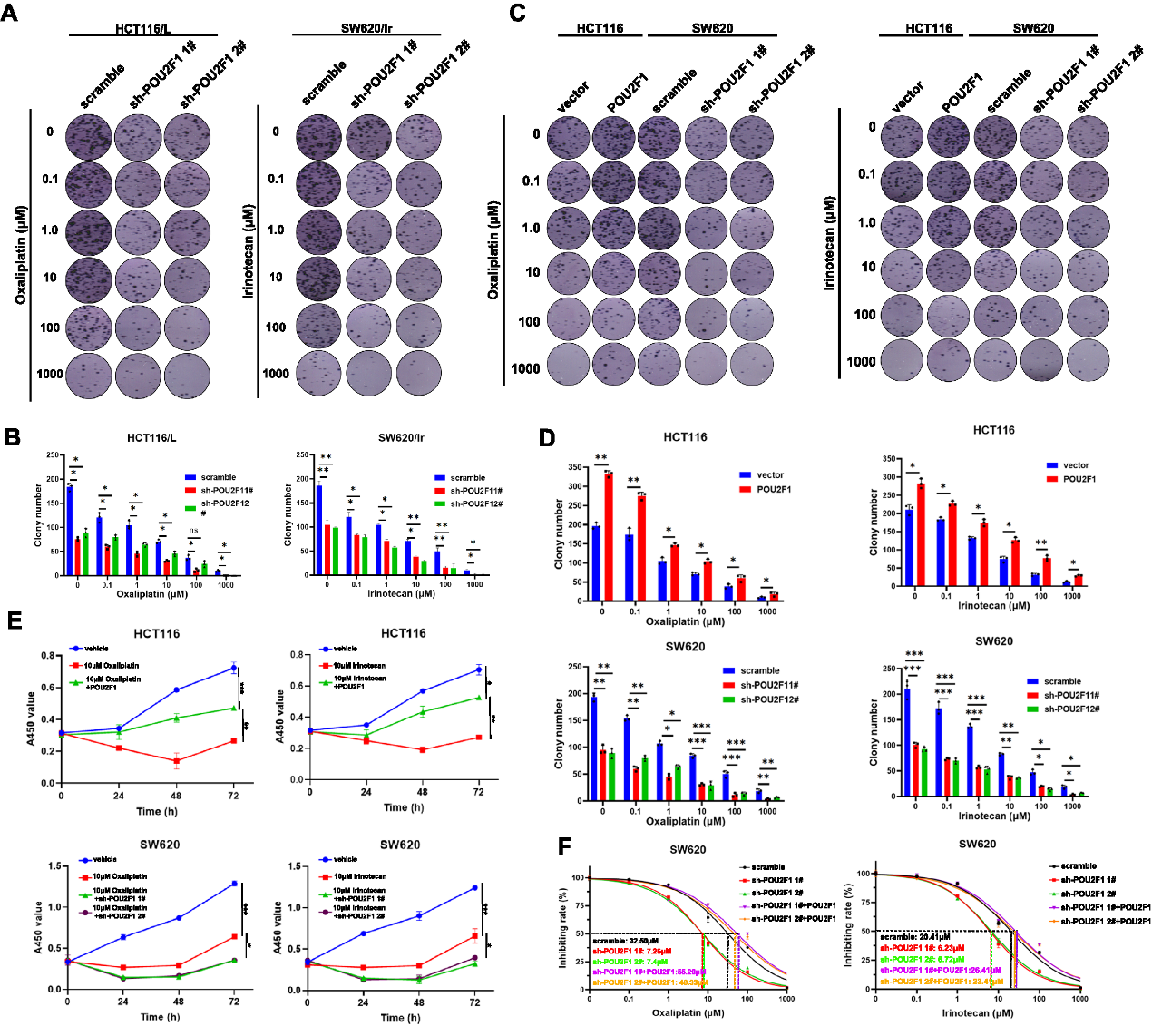


**Figure S2 POU2F1 promotes** **oxaliplatin and irinotecan resistance in CRC cells.**

(**A-D**) Colony formation assays to determine the influence of POU2F1 on oxaliplatin and irinotecan sensitivity in oxaliplatin-resistant HCT116 cells and irinotecan-resistant SW620 cells (**A**, **B**), and HCT116 and SW620 (**C**, **D**). (**E**) POU2F1 modulated the cell proliferation to 10μM oxaliplatin or irinotecan treatment in the indicated cells at 72 h post treatment. (**F**) Enforced POU2F1 expression rescued the oxaliplatin and irinotecan resistance of POU2F1-silencing SW620 cells.

**
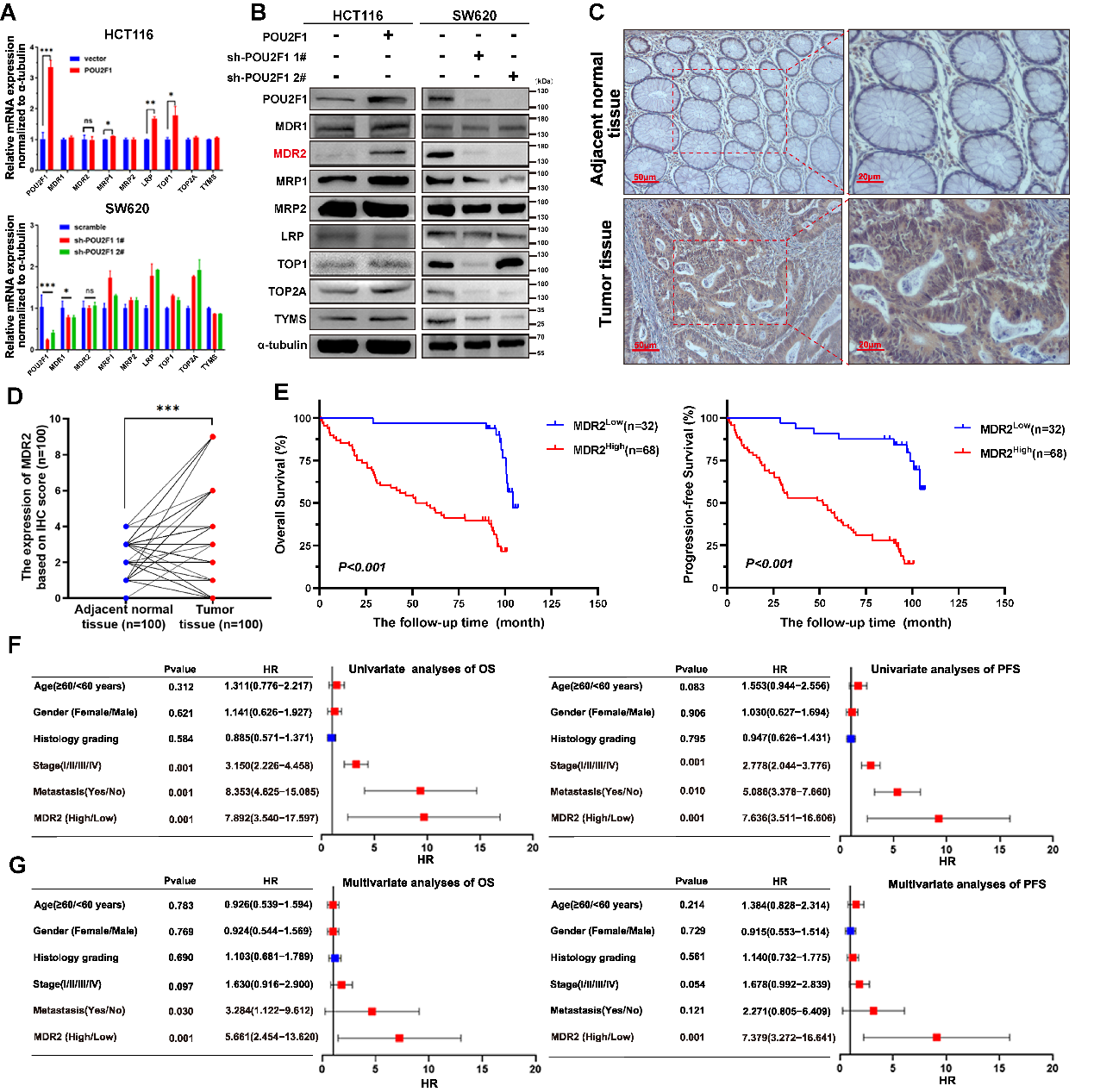
**

**Figure S3 Up-regulated MDR2 expression is associated with worse prognosis of colorectal cancer.**

(**A**, **B**) RT-qPCR and Western blotting analyses indicated the relative levels of eight resistance-related proteins in the indicated cells. (**C**) IHC analyses of MDR2 expression in adjacent normal tissues and colorectal cancer specimens (magnification x200, scale bars 50 μm, magnification x400, scale bars 20 μm). (**D**) Scatter plot exhibited the levels of MDR2 protein in 100 pairs of colorectal cancer tissues. (**E**) Kaplan-Meier analysis of OS and PFS in colorectal cancer patients after stratification with the median value of MDR2 protein in 100 pairs of colorectal cancer tissues. (**F**, **G**) Univariate (**F**) and multivariate (**G**) analyses of factors for the PFS and OS of colorectal cancer patients. Data are representative images or expressed as the mean ± SD of each group of samples analyzed in triplicate from three separate experiments. *P<0.05, **P<0.01, ***P<0.001, ns, no significance.

**
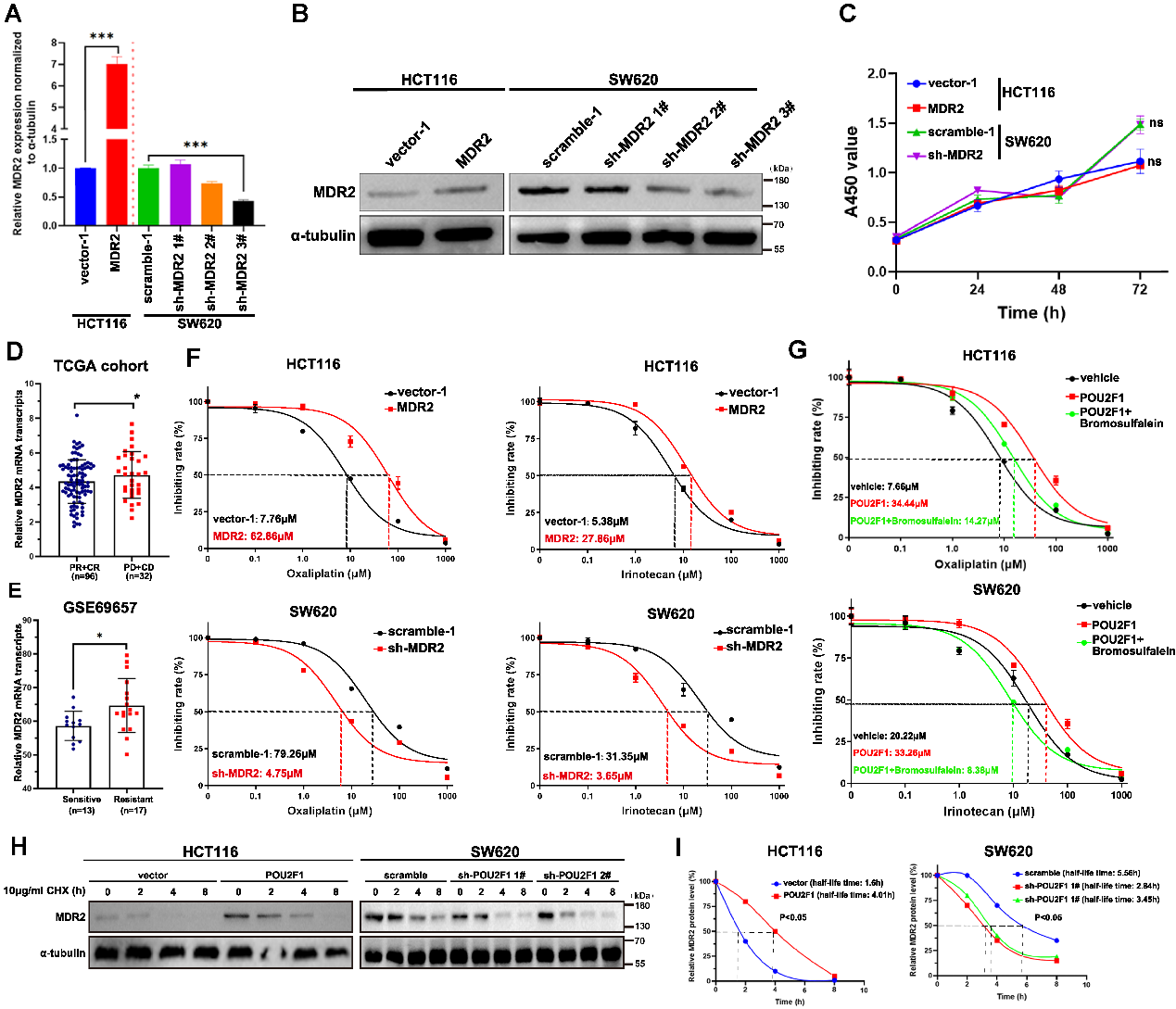
**

**Figure S4 POU2F1 induces oxaliplatin and irinotecan resistance via increasing MDR2 expression.**

(**A**, **B**) RT-qPCR and Western blot assays to verify the mRNA transcripts and protein expression of MDR2 in HCT116 and SW620 cell. (**C**) Cell proliferation of CRC cells following MDR2 shRNA and overexpressing transfection. (**D**) Scatter plot displayed the levels of MDR2 mRNA transcripts in COAD patients with PR/CR (n=96) compared with patients with PD/SD (n=32) after primary therapy based on TCGA cohort. (**E**) Plot chart comparing the expression of MDR2 in sensitive CRC tissues (n=13) compared with resistant tissues (n=17) in GSE69657 dataset. (**F**) Survival curves for the indicated groups of CRC cells treated with MDR2 overexpression/ silencing followed by oxaliplatin and irinotecan, respectively. (**G**) Survival curves for the indicated groups of CRC cells treated with or without 20µM Bromosulfalein treatment followed by oxaliplatin and irinotecan, respectively. (**H**, **I**) Cycloheximide (CHX)-chasing assay showing the levels of MDR2 proteins at indicated time points after treatment with 10μg CHX. Quantification results are shown on the right. Data are representative images or expressed as the mean ± SD of each group of samples analyzed in triplicate from three separate experiments. *P<0.05, **P<0.01, ***P<0.001, ns, no significance.


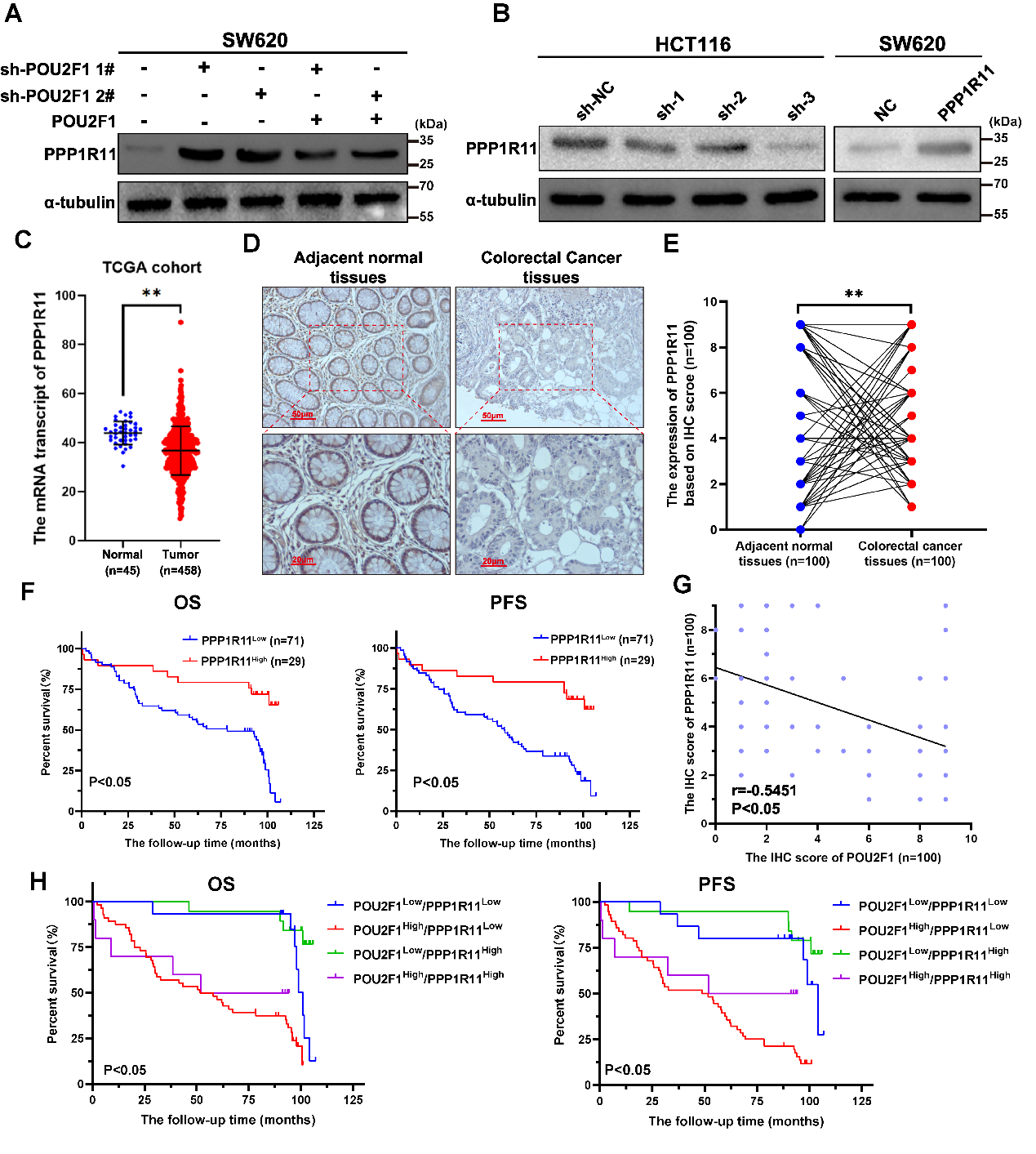


**Figure S5 Up-regulated MDR2 expression is associated with worse prognosis of colorectal cancer.**

(**A**) Western blot assays to verify the protein expression of PPP1R11 in the indicated cells. (**B**) Western blot assays to determine the protein expression of PPP1R11 in HCT116 and SW620 cells. (**C**) Scatter plot displayed the levels of PPP1R11 mRNA transcripts in 458 colon cancer (Tumors) and 45 non-tumor colon tissues (Normal) in TCGA cohort. (**D**, **E**) IHC analysis and Scatter plot of PPP1R11expression in 100 paired of colorectal cancer tissues (magnification x200, scale bars 50 μm, magnification x400, scale bars 20μm). (**F**) Kaplan-Meier analysis of OS and PFS in CRC patients after stratification with the median value of PPP1R11 based on IHC staining scores. (**G**) The correlation between the relative levels of POU2F1 and PPP1R11 in 100 paired of CRC tissues based on IHC staining scores. (**H**) 100 colorectal cancer patients were stratified into the POU2F1^Low^/PPP1R11^Low^ (n=15), POU2F1^High^/PPP1R11^Low^ (n=56), POU2F1^Low^/PPP1R11^High^ (n=19), and POU2F1^High^/PPP1R11^High^ (n=10). Based on the levels of POU2F1 and PPP1R11 expression by IHC. Data are representative images or expressed as the mean ± SD of each group of samples analyzed in triplicate from three separate experiments. **P<0.01.


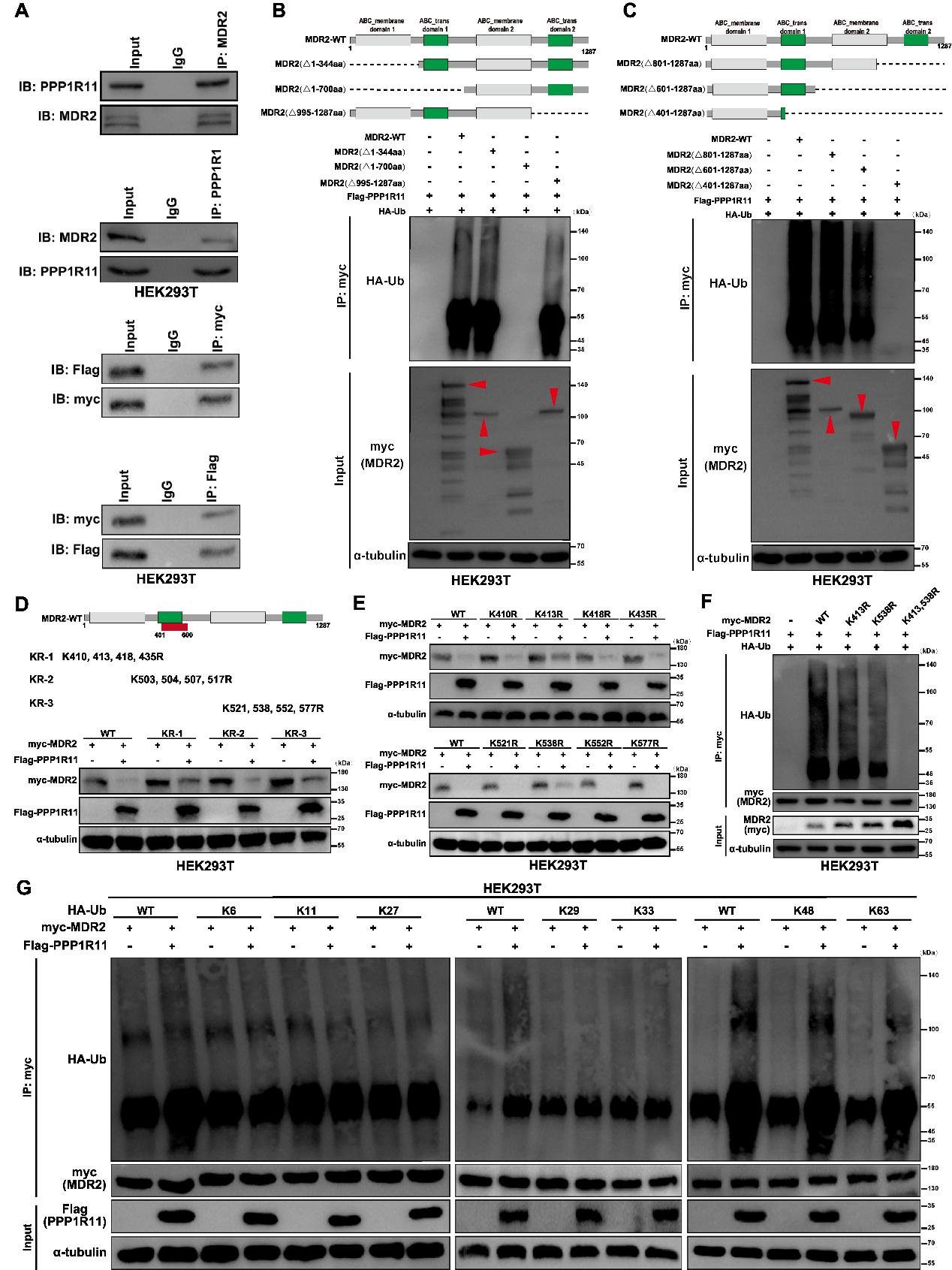


**Figure S6 PPP1R11 mediates the ubiquitination and degradation of MDR2.**

(**A**) IP analyses for endogenous and exogenous PPP1R11 and MDR2 in HEK293T cells or with myc-MDR2 and Flag-PPP1R11 transfection (lower panels). (**B, C**) Schematics for the structural domains of MDR2 and the designed mutants for mapping the ubiquitin aera to PPP1R11. The ubiquitination of MDR2 was measured using an *in vivo* ubiquitination assay. Cells were treated with MG132 for 4h before harvest. (**D**, **E**) Three groups KR (lysines that mutated to Arginines) mutations of myc-MDR2 was co-transfected with Flag-PPP1R11 in HEK293T cells, and the indicated proteins were detected by western blot. Lysine mutations in group KR-1 and KR-3 but not KR-2 abolished PPP1R11-mediated MDR2 degradation. (**F**) Mutation of K413 and K538 abolishes PPP1R11-mediated MDR2 degradation. Flag-PPP1R11 was co-transfected with wild-type or K413R and K538R mutant MDR2 and HA-Ub. The ubiquitination of MDR2 was measured using an *in vivo* ubiquitination assay. Cells were treated with MG132 for 4 h before harvest. (**G**) An *in vitro* ubiquitylation assay was performed as in using lysine-to-arginine Ub mutants (30μg: panel). The products were immunoblotted using anti-Ub antibody.


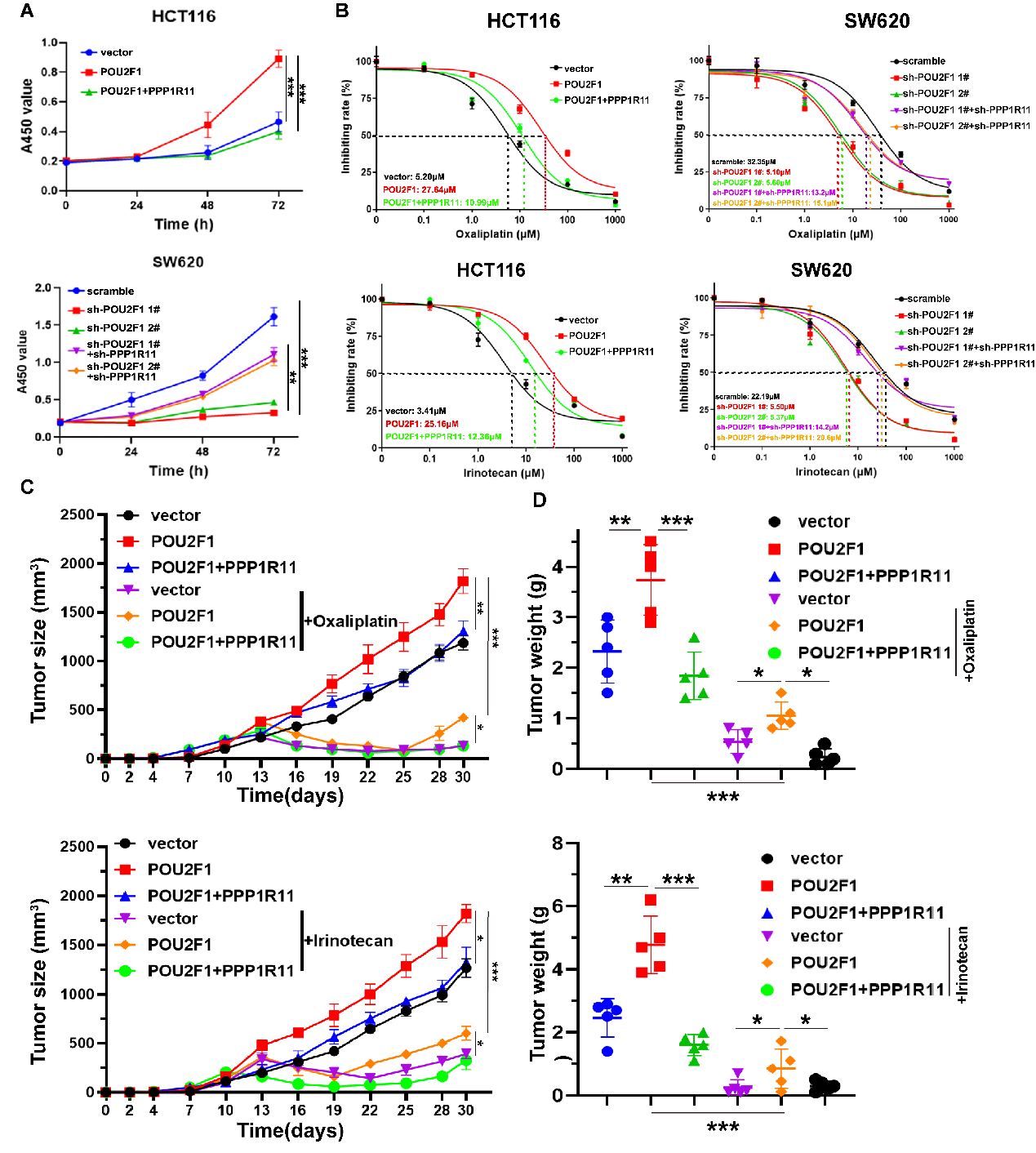


**Figure S7 POU2F1 induces chemoresistance by inhibiting PPP1R11 expression.**

(**A**) CCK8 assays of the proliferation ability in CRC cells treated with POU2F1 silencing/ overexpressing for 48h following PPP1R11 inhibition or overexpression, respectively. (**B**) Survival curves for the indicated groups of CRC cells treated with oxaliplatin and irinotecan, respectively. (**C**, **D**) Growth curves (**C**) and weight (**D**) of POU2F1‐overexpressing HCT116 following PPP1R11 overexpression xenograft tumors (n=5) after oxaliplatin and irinotecan treatment, respectively. Data are representative images or expressed as the mean ± SD of each group of samples analyzed in triplicate from three separate experiments. *P<0.05, **P<0.01, ***P<0.001, ns, no significance.


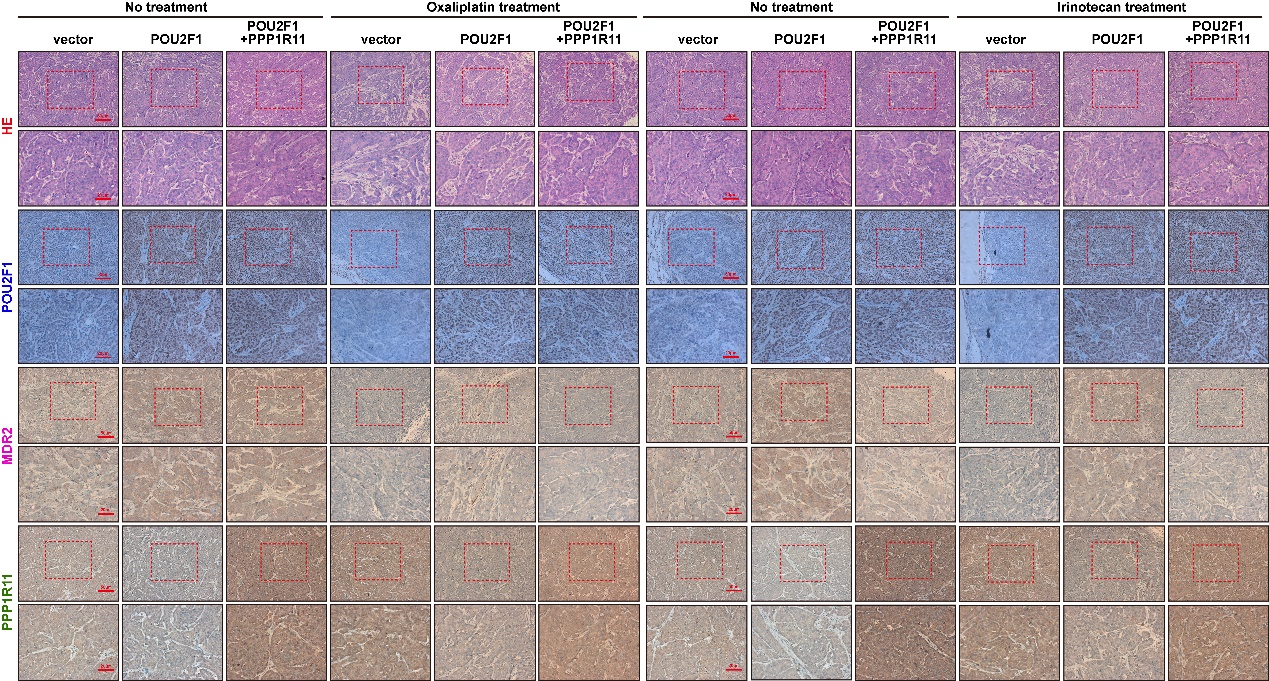


**Figure S8 POU2F1 inhibits PPP1R11 expression *in vivo*.**

Hematoxylin and eosin (H&E) and immunohistochemistry (IHC) analysis of POU2F1, MDR2 and PPP1R11 in xenograft tumors after oxaliplatin and irinotecan treatment, respectively.


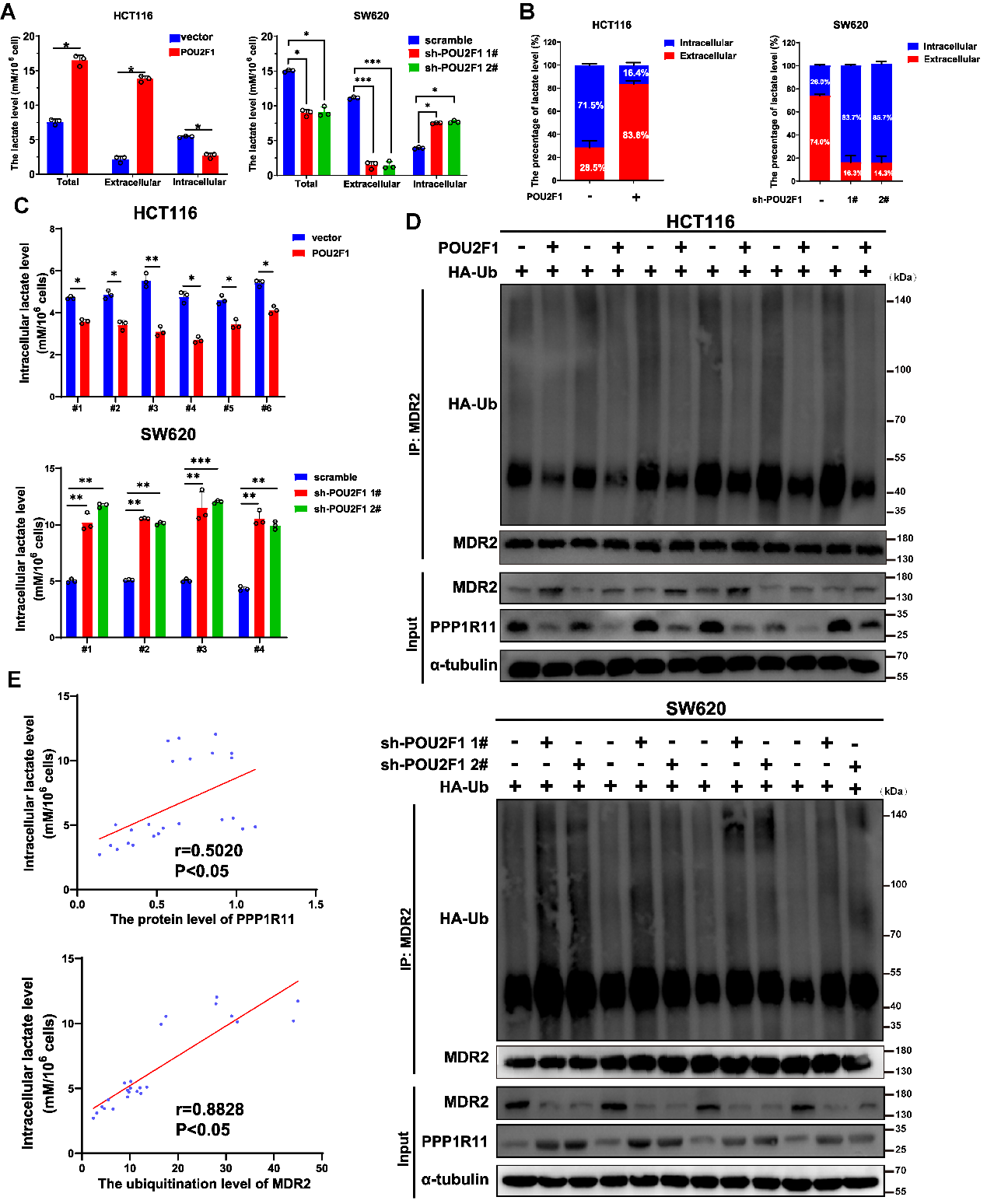


**Figure S9 The** **cytosolic lactate was positively associated with PPP1R11 expression and MDR2 ubiquitination.**

(**A**, **B**) Quantification (**A**) and percentage (**B**) of intracellular and extracellular L-lactate concentrations in the indicated cells using a lactate assay kit. (**C**) Measurement of cytosolic lactate in the indicated cells. (**D**) Western blotting analysis of PPP1R11 expression and MDR2 ubiquitination in CRC cells with stable POU2F1 overexpression/ inhibition. (**E**) The correlation between cytosolic lactate level and PPP1R11 protein levels and MDR2 ubiquitination as described in A-B. Data are representative images or expressed as the mean ± SD of each group of samples analyzed in triplicate from three separate experiments. *P<0.05, **P<0.01, ***P<0.001.


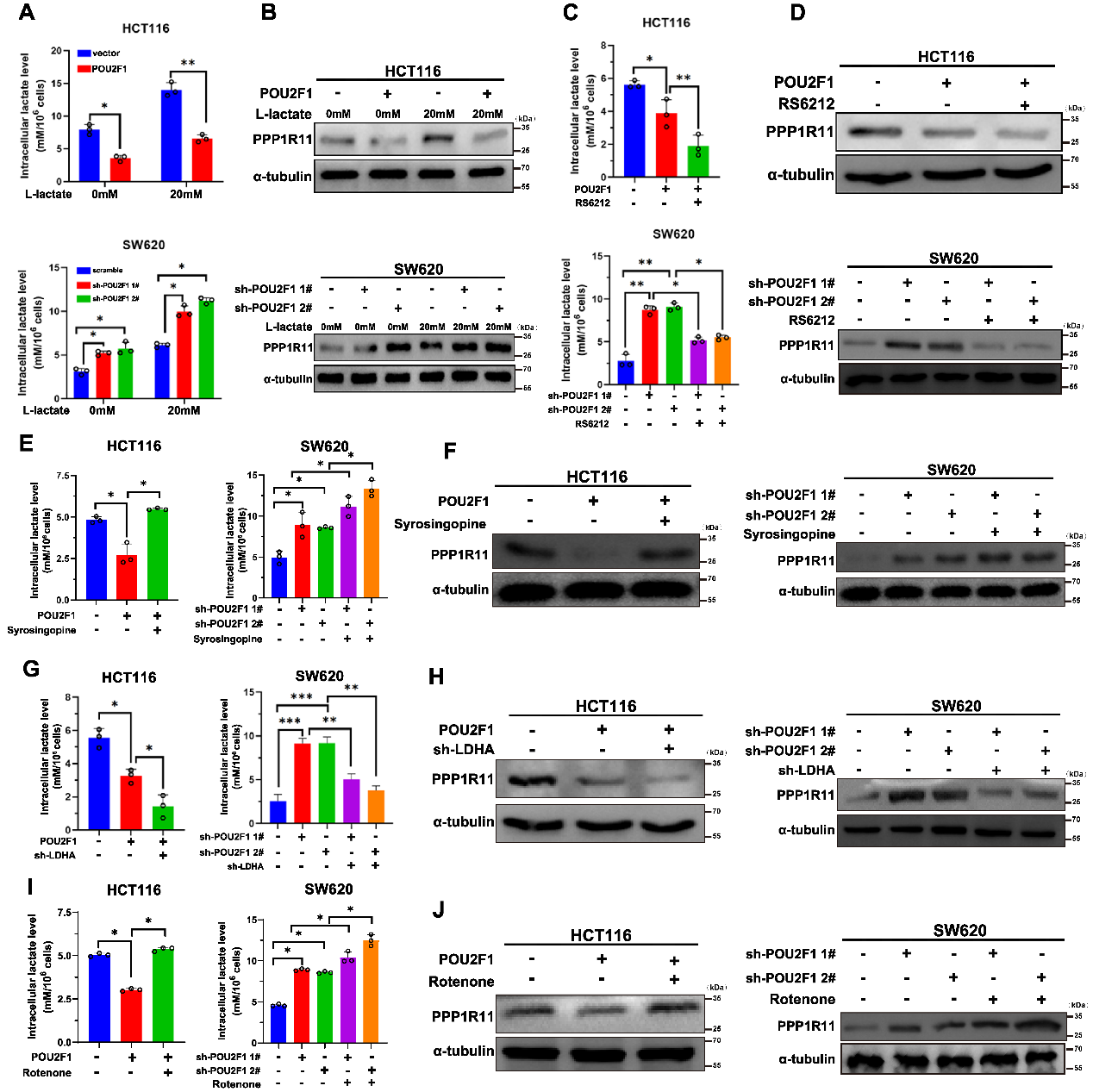


**Figure S10 POU2F1 induces PPP1R11 inhibition via decreasing intracellular lactate level.**

(**A**, **B**) Measurement of cytosolic lactate (**A**) and PPP1R11 expression (**B**) by western blotting in the indicated cells with or without 20mM lactate treatment. (**C**-**J**) Measurement of cytosolic lactate (**C**, **E**, **G**, **I**) and PPP1R11 expression (**D**, **F**, **H**, **J**) by western blotting in the indicated cells with or without 10mM RS6212 (a specific inhibitor of LDH) (**C**, **D**), 20μg/ml Syrosingopine treatment (**E**, **F**), siRNAs for LDHA expression (**G**, **H**), or 50mM rotenone treatment (**I**, **J**). Data are representative images or expressed as the mean ± SD of each group of samples analyzed in triplicate from three separate experiments. *P<0.05, **P<0.01, ***P<0.001.

**
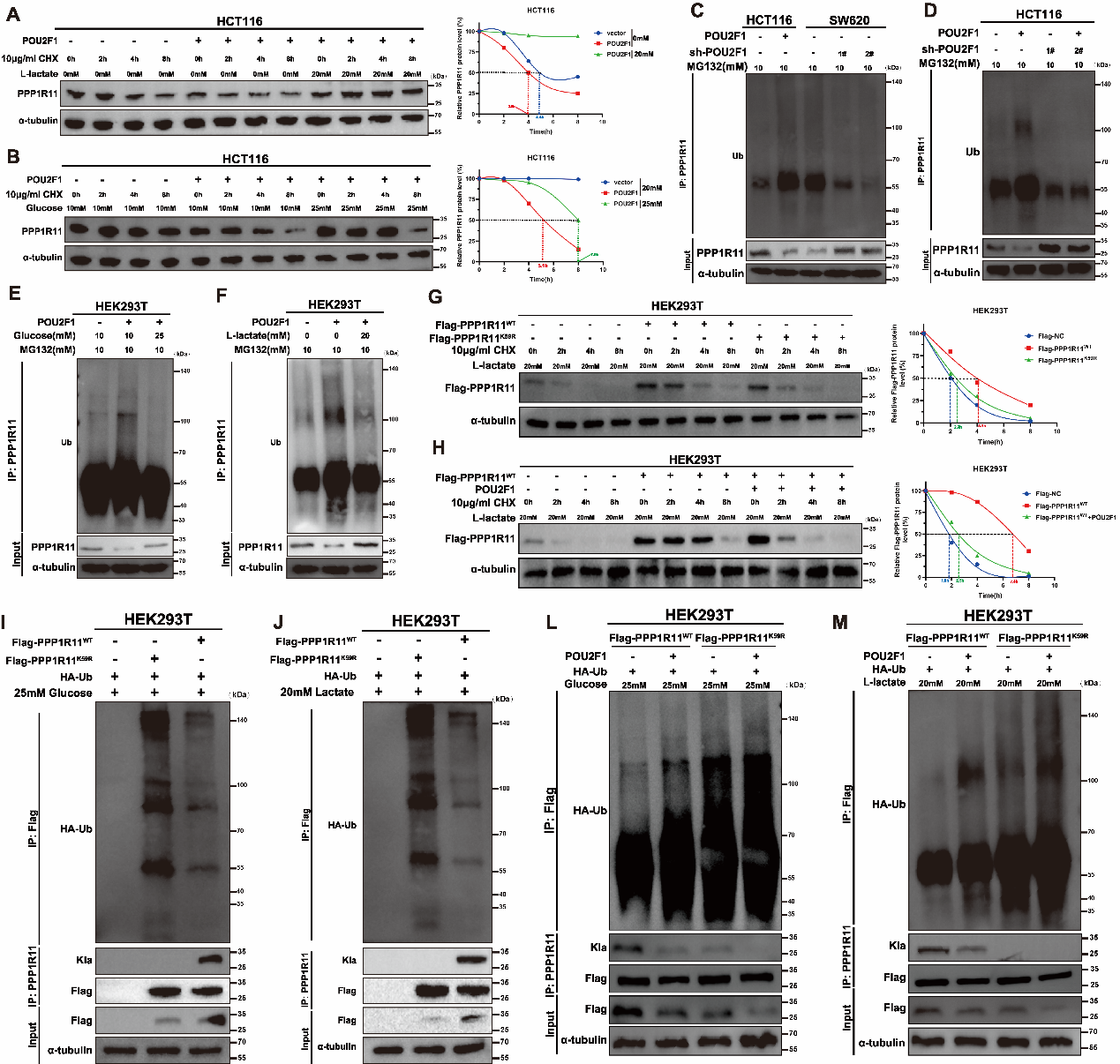
**

**Figure S11 PPP1R11 lactylation contributes to maintaining its stability.**

(**A**, **B**) Cycloheximide (CHX)-chase assay showing the degradation of PPP1R11 in HCT116 cells treated with POU2F1 expression stimulated with 20 mM lactate (**A**) and 10 mM/25 mM glucose treatment (**B**). (**C**) *In vivo* ubiquitination of PPP1R11 in POU2F1 silencing/ overexpressing CRC cells treated with 10mM MG132. (**D**) *In vivo* ubiquitination of PPP1R11 in POU2F1 overexpressing HCT116 cells following POU2F1 silencing treated with 10mM MG132. (**E**, **F**) *In vivo* ubiquitination of PPP1R11 in POU2F1 overexpressing HEK293T cells treated with 10 mM/25 mM glucose (**E**) or 20 mM lactate (**F**) for 24 h. (**G**) Cycloheximide (CHX)-chase assay showing the degradation of PPP1R11^WT^ and PPP1R11^K59R^ in HEK293T cells treated with 20 mM lactate. (**H**) POU2F1 was co-transfected with Flag-tag wild-type of PPP1R11 in HEK293T cells treated with 20 mM lactate. The ubiquitination of Flag-PPP1R11 was measured using an *in vivo* ubiquitination assay. (**I**, **J**) wild-type or catalytic inactive mutant of Flag-PPP1R11 was co-transfected with HA-Ub in HEK293T cells treated with 25 mM glucose (**I**) or 20 mM lactate (**J**) for 24 h. The ubiquitination of PPP1R11 was measured using an *in vivo* ubiquitination assay. Cells were treated with MG132 for 4 h before harvest. (**L**, **M**) POU2F1 was co-transfected with wild-type or catalytic inactive mutant of PPP1R11 and HA-Ub in HEK293T cells treated with 25 mM glucose (**I**) or 20 mM lactate (**J**) for 24 h. The ubiquitination of PPP1R11 was measured using an *in vivo* ubiquitination assay. Cells were treated with MG132 for 4 h before harvest.


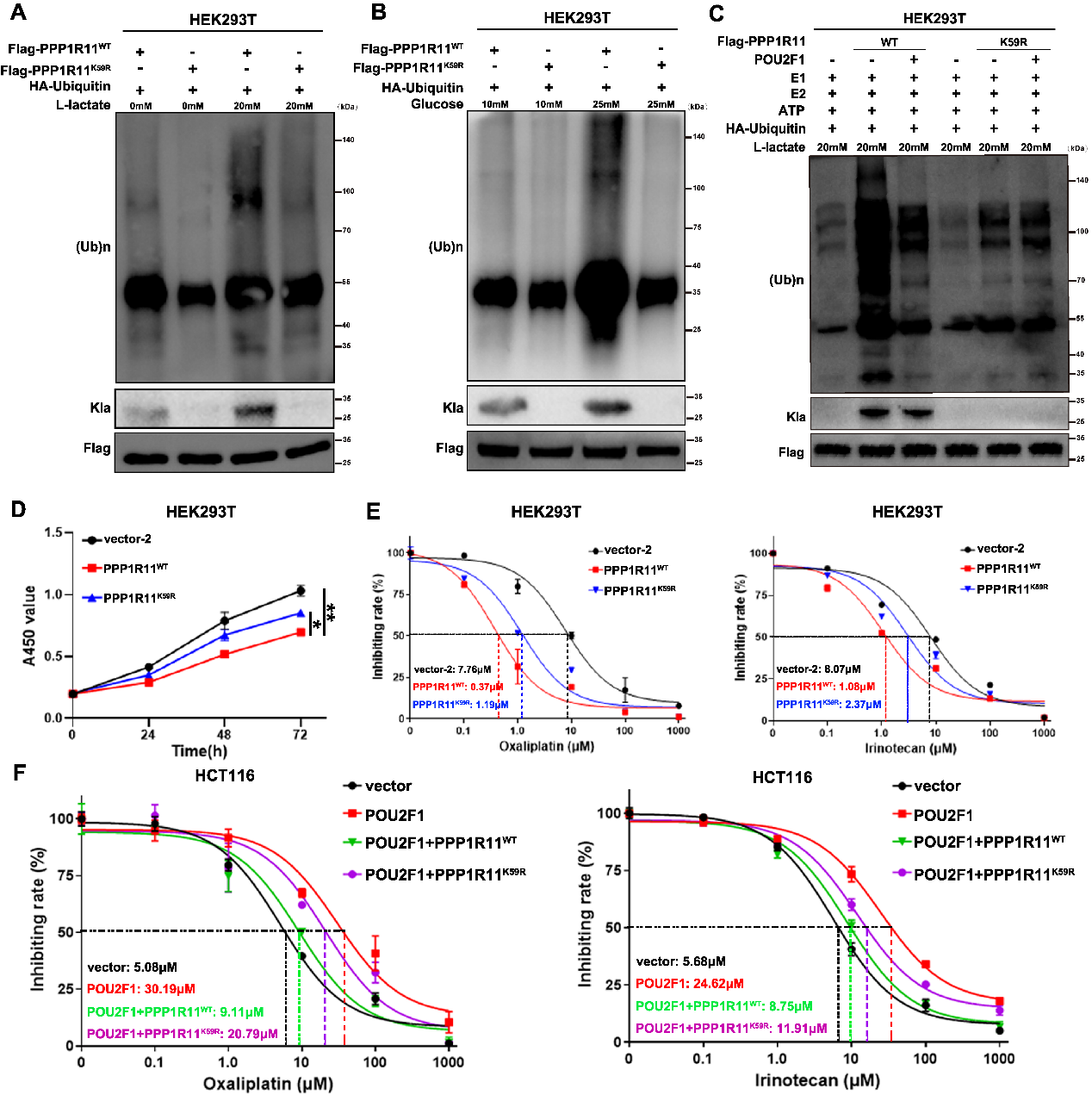


**Figure S12 PPP1R11 lactylation contributes to inhibiting CRC growth and chemoresistance.**

(**A**, **B**) The E3 Ligase activity of wild-type or catalytic inactive mutant of PPP1R11 in HEK293T cells treated with 20 mM lactate (**A**) or 25 mM glucose (**B**) for 24 h. (**C**) The E3 Ligase activity assay showing the impact of POU2F1 on wild-type or catalytic inactive mutant of PPP1R11 in HEK293T cells treated with 20 mM lactate for 24 h. (**D**) CCK8 assays of the proliferation ability in CRC cells transfected with wild-type or catalytic inactive mutant of PPP1R11. (**E**) HEK293T cells were transfected with PPP1R11^WT^ or PPP1R11^K59R^, and after 48 hours the cells were passed and treated with different concentrations of oxaliplatin and irinotecan. Cell survival rate was calculated and cell survival curve was drawn. (**F**) POU2F1 were co-transfected with PPP1R11^WT^ or PPP1R11^K59R^ in HCT116 cells, and after 48 hours the cells were passed and treated with different concentrations of oxaliplatin and irinotecan. Cell survival rate was calculated and cell survival curve was drawn. Data are representative images or expressed as the mean ± SD of each group of samples analyzed in triplicate from three separate experiments. *P<0.05, **P<0.01.


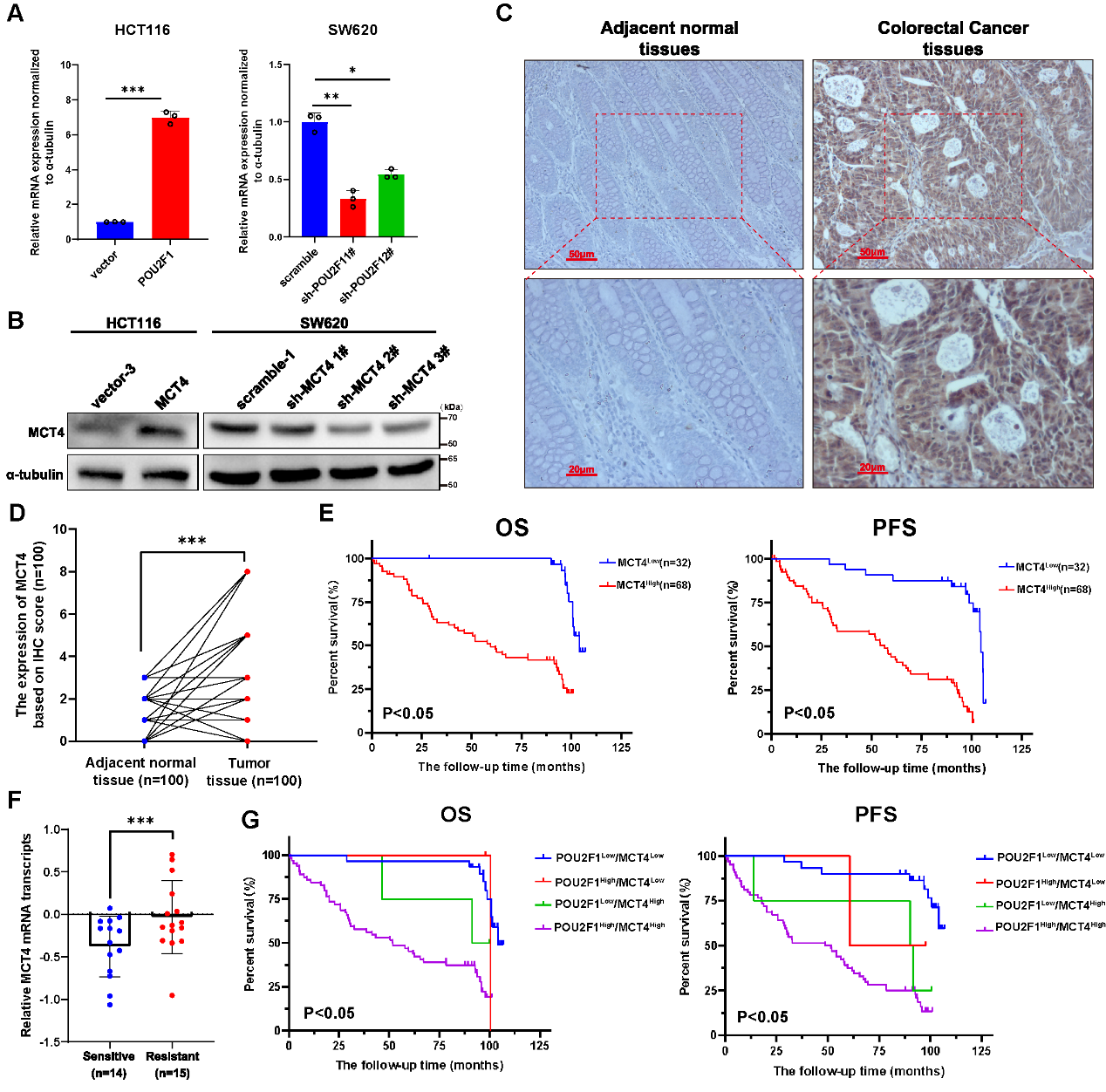


**Figure S13 POU2F1 attenuates PPP1R11 lactylation via decreasing MCT4 expression.**

(**A**, **B**) qRT-PCR assay and Western blotting analysis for detecting MCT4 mRNA (**A**) and protein expression (**B**) in CRC cells after POU2F1 overexpression and inhibition. (**C**) Kaplan-Meier analysis of OS and PFS in CRC patients after stratification with the median value of MCT4 based on IHC staining scores. (**D**) 100 colorectal cancer patients were stratified into the POU2F1^Low^/MCT4^Low^ (n=30), POU2F1^High^/MCT4^Low^ (n=2), POU2F1^Low^/MCT4^High^ (n=4), and POU2F1^high^/MCT4^high^ (n=64). Based on the levels of POU2F1 and MCT4 expression by IHC. *P<0.05, **P<0.01, ***P<0.001.


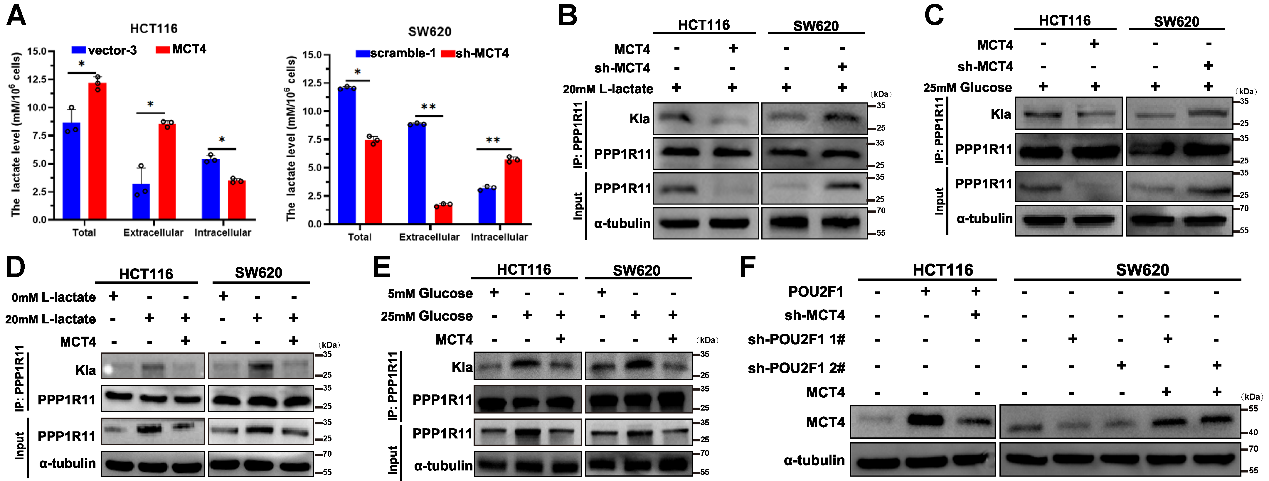


**Figure S14 POU2F1 attenuates PPP1R11 lactylation via decreasing MCT4 expression.**

(**A**) Measurement of total, intracellular and extracellular lactate in CRC cells after MCT4 overexpression and inhibition. (**B**, **C**) Western blotting analysis for detecting PPP1R11 expression and lactylation in the indicated cells treated with 20 mM lactate (**B**) or 25 mM glucose (**C**) for 24 h. (**D**, **E**) Western blotting analysis for detecting PPP1R11 expression and lactylation in the indicated cells treated with 20 mM lactate (**D**) or 10/25 mM glucose (**E**) for 24 h and then transfected with POU2F1 expression. (**F**) Western blotting analysis for detecting MCT4 expression in the indicated cells. Data are representative images or expressed as the mean ± SD of each group of samples analyzed in triplicate from three separate experiments. *P<0.05, **P<0.01, ***P<0.001.

**Supplementary Tables**

**Table S1. The sequences of primers for ChIP**

| **Sites** | **Forward (5'-3')** | **Reverse (5'-3')** |
| --- | --- | --- |
| Site 1 | AATGACTTGCTGTTCTGCCG | AGTGAATCATTGTGCCTAACCT |
| Site 2 | CTGGCTCCTTACAGTGGTTT | CACCACCATGCTCAGCTAAT |
| Site 3 | CAACAAGCGTGCCACCAAAA | GGCTGGTCTCAAACTCCTGA |
| Site 4 | TGGGCGACAGAGTGAGACT | AGCTTAGTGGTTTGGGAGGG |
| Site 5 | CCCTCCCAAACCACTAAGCT | TCTTTCGTCTCCCACACCAA |
| Site 6 | GGGTAGAGGGAGGAAATGGG | TGATTACCTCAACCACCTCCT |

**Table S2. The sequences of primers for RT-qPCR**

| **Genes** | **Refseq No.** | **Forward (5'-3')** | **Reverse (5'-3')** |
| --- | --- | --- | --- |
| POU2F1 | NM_001198783.2 | GATCTGCCTGGTGAATGCTG | ATCGCCGCAAAACATCTCTC |
| MDR1 | NM_001348945.2 | CAGCATTCAGTCAATCCGGG | TTCTTTGCTCCTCCATTGCG |
| MDR2 | NM_018850.3 | TGAAACTTATGGCTCCCCGT | AAGAAACGGATGCCCAGAGA |
| MRP1 | NM_004996.4 | TCAGCTCGTCTTGTCCTGTT | AAACAGGCACGACTTGTTCC |
| MRP2 | NM_000392.5 | CTATGTCCCACAGCAGTCCT | TCAGCCAAATCTCCTCCAGG |
| LRP | NM_001388321.1 | GATCCGACCAGTCAGAAGCC  GAG | AATTCACTTCTTCACCTCCAC  CTCAGCC |
| TOP1 | NM_003286.4 | AGACGGAAGCTCGGAAACAG | ATCCTCGGGCATGATTCGTC |
| TOP 2A | NM_001067.4 | ACAGGTGGTCGAAATGGCTA | TCTTTGTCCAGGCTTTGCAT |
| TYSM | NM_001354867.2 | ATCATGTGCGCTTGGAATCC | CGCAATCATGTACGTGAGCA |
| S MCT2 | NM_178498.4 | CTGCCAGGACTTTTTGTGGC | GAGGCTGGTGCAGGGTAAAT |
| MCT3 | NM_001394131.1 | ACATACCTGCTCCACTTCCC | CCAAGTCTCCTTCTCCTGCA |
| MCT4 | NM_001201548.2 | GATATGGGCGCTTACCATTTTCG | TGTGCTGCGTGACATTCCAA |
| α-tubulin | NM_006009.4 | CACCATCAAAACCAAGCGCA | GCAGGGCCAAAAGGAATGGA |

**Table S3. Antibodies for Western blotting, immunofluorescence, and immunohistochemistry**

| **Antibody** | **Catalog number** | **Company** | | **Experiment** | **Dilution** |
| --- | --- | --- | --- | --- | --- |
| anti-POU2F1 | #8157 | CST | Western Blotting | | 1:500 |
|  |  |  | Immunofluorescence | | 1:100 |
|  |  |  | Immunohistochemistry | | 1:50 |
|  |  |  | ChIP | | 1:10 |
| anti-PPP1R11 | ab171960 | Abcam | Western Blotting | | 1:500 |
|  |  |  | Immunohistochemistry | | 1:50 |
|  |  |  | Immunoprecipitation | | 1:50 |
| anti-MDR2 | PA5-78692 | Invitrogen | Western Blotting | | 1:500 |
|  |  |  | Immunohistochemistry | | 1:50 |
|  |  |  | Immunofluorescence | | 1:50 |
| anti-MDR1 | #13978 | CST | Western Blotting | | 1:500 |
| anti-MRP1 | #72202 | CST | Western Blotting | | 1:500 |
| anti- MRP2 | #4446 | CST | Western Blotting | | 1:400 |
| anti-LRP | ab273093 | Abcam | Western Blotting | | 1:100 |
| anti-TOP 1 | #79971 | CST | Western Blotting | | 1:500 |
| anti-TOP 2A | ab52934 | Abcam | Western Blotting | | 1:500 |
| anti-TYMS | #9045 | CST | Western Blotting | | 1:500 |
| anti-α-tubulin | #2144 | CST | Western Blotting | | 1:1000 |
| anti-His | #12698 | CST | Western Blotting | | 1:500 |
|  |  |  | Immunoprecipitation | | 1:50 |
| anti-Flag | MA1-91878 | Invitrogen | Western Blotting | | 1:500 |
|  |  |  | Immunoprecipitation | | 1:50 |
| anti-myc | ab32 | Abcam | Western Blotting | | 1:500 |
|  |  |  | Immunoprecipitation | | 1:50 |
| anti-SMCT2 | ab262934 | Abcam | Western Blotting | | 1:500 |
| anti-MCT3 | PA5-115908 | Invitrogen | Western Blotting | | 1:500 |
| anti-MCT4 | ab191008 | Abcam | Western Blotting | | 1:500 |
| anti-Ubiquitin | ab134953 | Abcam | Western Blotting | | 1:500 |
| anti-HA | #3724 | CST | Western Blotting | | 1:500 |
|  |  |  | Immunoprecipitation | | 1:50 |
| anti-Kla | PTM-1401RM | PTM Bio | Western Blotting | | 1:500 |
